# Characteristics of mental health recovery narratives: Systematic review and narrative synthesis

**DOI:** 10.1371/journal.pone.0214678

**Published:** 2019-03-28

**Authors:** Joy Llewellyn-Beardsley, Stefan Rennick-Egglestone, Felicity Callard, Paul Crawford, Marianne Farkas, Ada Hui, David Manley, Rose McGranahan, Kristian Pollock, Amy Ramsay, Knut Tore Sælør, Nicola Wright, Mike Slade

**Affiliations:** 1 School of Health Sciences, Institute of Mental Health, University of Nottingham, Nottingham, United Kingdom; 2 Department of Psychosocial Studies, Birkbeck University, London, United Kingdom; 3 College of Health and Rehabilitation Sciences, Boston University, Boston, Massachusetts, United States of America; 4 Nottinghamshire Healthcare NHS Foundation Trust, Nottingham, United Kingdom; 5 Unit of Social and Community Psychiatry, Blizard Institute, Barts and the London School of Medicine and Dentistry, Queen Mary University of London, London, United Kingdom; 6 School of Health Sciences, University of Nottingham, United Kingdom; 7 Health Service and Population Research Department, Institute of Psychiatry, Psychology and Neuroscience, King's College London, London, United Kingdom; 8 Faculty of Health and Social Sciences, Department of Health, Social and Welfare Studies, Center for Mental Health and Substance Abuse, University of South Eastern Norway, Kongsberg, Norway; Maastricht Universitair Medisch Centrum+, NETHERLANDS

## Abstract

**Background:**

Narratives of recovery from mental health distress have played a central role in the establishment of the recovery paradigm within mental health policy and practice. As use of recovery narratives increases within services, it is critical to understand how they have been characterised, and what may be missing from their characterisation thus far. The aim of this review was to synthesise published typologies in order to develop a conceptual framework characterising mental health recovery narratives.

**Method:**

A systematic review was conducted of published literature on the characteristics of mental health recovery narratives. Narrative synthesis involved identifying characteristics and organising them into dimensions and types; and subgroup analysis based on study quality, narrator involvement in analysis, diagnosis of psychosis and experience of trauma. The synthesis was informed by consultation with a Lived Experience Advisory Panel and an academic panel. The review protocol was pre-registered (Prospero CRD42018090188).

**Results:**

8951 titles, 366 abstracts and 121 full-text articles published January 2000-July 2018 were screened, of which 45 studies analysing 629 recovery narratives were included. A conceptual framework of mental health recovery narratives was developed, comprising nine dimensions (Genre; Positioning; Emotional Tone; Relationship with Recovery; Trajectory; Use of Turning Points; Narrative Sequence; Protagonists; and Use of Metaphors), each containing between two and six types. Subgroup analysis indicated all dimensions were present across most subgroups, with Turning Points particularly evident in trauma-related studies.

**Conclusions:**

Recovery narratives are diverse and multidimensional. They may be non-linear and reject coherence. To a greater extent than illness narratives, they incorporate social, political and rights aspects. Approaches to supporting development of recovery narratives should expand rather than reduce available choices. Research into the narratives of more diverse populations is needed. The review supports trauma-informed approaches, and highlights the need to understand and support post-traumatic growth for people experiencing mental health issues.

## Introduction

Recovery has become the established orientation within mental health policy and provision at national [1] and international [2] levels. It has been defined as “a deeply personal, unique process of change… a way of living a satisfying, hopeful and contributing life even with limitations caused by illness [and] a process involving the development of new meaning or purpose in one’s life” ([3] p. 527).

Knowledge about recovery is based primarily on the individual stories and resulting insights of those with lived experience of psychological distress [4]. The sharing of individual stories was central to the psychiatric survivor and user-led movements which originated in the 1960s and 1970s [5]. Stories of psychological distress and recovery emphasised empowerment and self-determination [6]. Heard collectively, they enabled survivors and users to build solidarity and inspire hope in the face of widespread stigma, discrimination and denial of rights [7]. Recovery has thus been framed as a civil rights movement [8], and mirrors other forms of identity politics in which (re)claiming a voice to author one’s own story is a central emancipating act. A core preoccupation of Mad Studies, the academic discipline which has emerged around the survivor/service user movement, is correspondingly the production of knowledge based on collective discourses of direct experience of madness [9].

Sharing experiences through stories has become a central practice within recovery-based healthcare [10]. Story-sharing has been used to increase the empathy and understanding of healthcare staff [11] and as a mechanism of peer support [12]. Sharing one’s recovery story was identified as a unique task for peer support workers in a U.S. national survey [13]. It offers a means of enabling individuals to make sense of their experience and feel heard by others, through for example the widely-offered “Telling Your Story” course at UK Recovery Colleges [14]. The right and ability to define one’s own experiences and externalise negative dominant discourses is described by Onken and colleagues [15] as the primary mechanism in recovery. A recent systematic review has produced a conceptual framework of the impacts of recovery narratives on recipients, identifying five types of impact: connectedness; understanding of recovery; reduction in stigma; validation of personal experience; and emotional and behavioural responses [16]. Personal stories have thus been described as a key “recovery technology”, both embodying the values associated with recovery and providing a means of realising those values [17].

The terms *stories* and *narratives* are often used interchangeably, but following Smith and Sparkes [18] the current review uses *stories* to refer to the actual tales people tell, and *narratives* when considering the dimensions and properties comprising particular stories, for example within the research context. Research interest in narratives grew rapidly in the late twentieth-century ‘narrative turn’ within the social sciences [19]. Narrative medicine, for example, moved away from research methods drawing on logical proof and empirical observation towards methods capable of accessing experiential knowledge. Analysis of individual narratives offered new insights into how people interpret their worlds and assign meaning to their experiences; namely, the kind of cognitive, affective and aesthetic processes not available through direct observation or traditional forms of medical enquiry, yet central to illness and recovery experiences [20].

Narratives can be differentiated into internal narratives, told to oneself in response to events, and externally expressed narratives, told for and perhaps elicited by others [21]. The focus of the current review is on stories told for others. As “presentations of the self” [22], such narratives are subject to influences such as social desirability [23], and can be seen as resources employed by narrators, consciously or not, to frame or shape identities within particular contexts [24].

Within health research, interest in personal narratives has translated into the investigation of ‘illness narratives’ as a key source of understanding the experiences of people with chronic conditions, including cancer [25], HIV and AIDS [26], arthritis [27] and diabetes [28]. The experience of illness can be seen as a “call for stories” in literal and metaphorical ways ([25] p.53). When someone is ill, they are required literally to account for what is happening to healthcare professionals, employers, family and friends. Metaphorically, illness can be seen as a profound interruption to a person’s sense of self, making “narrative wreckage” of the previously expected trajectory of life ([25] p.110). An opportunity to undertake “life story work” [29], whether taken on personally, more formally within therapeutic services or simply through informal interaction, for example with healthcare staff, can be seen a means of restoring “prized” identities within the context of illness [30].

Types of illness narrative have been researched, for example Frank’s differentiation of restitution, chaos and quest narratives [25]. These types identify differing patterns of meaning that narrators may form from their experiences and, in the case of narratives told for others, from the (perceived) requirements of different audiences. Frank’s types have been highly influential within the study of both illness and recovery narratives, as explored below. Frank has also been critiqued along with other narrative theorists for assuming an innate narrative drive in humans, and for promoting particular kinds of narrative as “transcultural, trans-historical truths” instead of acknowledging their cultural specificity ([31], p.2).

Within mental health-related research, narrative approaches have equated mental illness with the breakdown of an individual’s coherent life story, whether as an inherent aspect of mental illness [21], or as a response to traumatising events [32]. The privileging of such qualities as coherence within narrative has also been challenged [33]; as has the assumed inability of people with, for example, experiences of psychosis to construct a coherent narrative [34]. Contrasting ways of approaching narratives within psychotherapeutic research and practice have thus been developed.

Therapists working within psychodynamic approaches may view listening to narratives as a way of gaining access to other areas of experience, such as relationship themes or other unconscious content [35]. These approaches have been criticised for invoking the telling of “deficit” narratives [36] and positioning the therapist as expert, a “vision of human possibility as unattainable as the heroism of cinematic mythology” ([37], p.171).

Social constructionists adopt what may be a more emancipatory perspective, viewing narratives themselves as the central focus. A sense of identity, in this approach, is not discovered in but actively constructed by the act of telling stories [38], with the construction of personal narratives providing opportunities to change an individual’s view of reality [39]. Therapeutic interventions derived from these approaches, such as narrative therapy, seek to help people resolve or reframe their emotional distress by constructing new types of stories [40], with therapist as witness, director, editor, interpreter or co-author [35]. Within constructionist approaches, stories can be seen as individual renderings of broader cultural narratives. And since “we do not tell stories about ourselves under conditions of our own choosing” ([41] p.6), the types of narrative available in any given historical moment are crucial in terms of the choices available to the narrator [42]. The implications of this are explored by the current review.

In the 1990s, mental health research studies began to focus specifically on the recovery aspects of illness narratives (see, for example, [43]). Influenced by survivor/user movements, Mad Studies and survivor/user-led research, researchers began actively to seek out narratives of recovery [44], both mirroring and further enabling the paradigm shift towards recovery in policy and practice. Contrasting approaches within studies of recovery narratives have emerged, similar to those within psychotherapeutic research and practice: those which use narratives as a means of accessing other information, and those investigating aspects of the narratives themselves.

Studies using narratives as a means of accessing other information take a broadly thematic approach. These studies are concerned with the content of participants’ narratives, and what can be learned about recovery from them (see, for example, [45]]. The objectives here are of increasing academic and clinical understanding of the nature of recovery [46] and providing a source of knowledge and hope for survivors [47]. A systematic review of Narrative Inquiry studies (n = 4) identified four higher-order concepts: recovery is possible; recovery is a journey; being in control of your own recovery is crucial; and the role of community in recovery [7]. The aim of the review was to elevate the expertise of people with lived experience to stand alongside quantitative findings, seen as the kind of evidence required to influence clinical policy and practice.

A second approach to recovery narratives is broadly structural, assuming that the way individuals narrate their experiences, and the kinds of narratives they construct, can also offer important understanding of recovery. These studies investigate various characteristics of recovery narratives, for example types of genre and tone [48], trajectories [49] or ways in which recovery narratives are positioned in relation to the dominant clinical narrative [50]. No systematic review of these studies has yet been undertaken, and this provides the rationale for the current review. Before considering the aims of this study, however, it is important to address both the benefits and potential risks of characterising narratives.

Analysis of narratives within research have played a central role in establishing the recovery orientation in practice. Narratives and recovery can be seen as “sister paradigms” in their ontological, epistemological and methodological overlaps [10]. However, research on narratives does not necessarily have a benign effect. Studies identifying types of recovery narrative may seem to suggest that only certain kinds of narratives exist, or are acceptable. This may contribute to a “narrowing of narrative templates” [51]. The risk is that conventional clinical narratives are simply replaced with new “dominant recovery narratives”, as a recent investigation of narrative-based interventions at UK Recovery Colleges has highlighted ([14], p.26). Such interventions may inadvertently constrain the kinds of story that individuals feel they can tell within these contexts, and may “needlessly and wrongly distress those who do not fit their model” ([31], p.77).

Narrative approaches also contain potential or implicit bias, due for example to the wider availability within research of written narratives from high-income or Anglophone countries. Other approaches and concepts may therefore be occluded in these contexts, for example those in the global South, or indigenous knowledge. Any approach to characterising narratives of distress and/or recovery should take this into account and acknowledge that templates may not be applicable universally, as noted by Woods [31] in her exploration of the limits of narrative.

With these caveats in place, characterising mental health recovery narratives may have many potential uses. For example, collective approaches to narratives add value by providing sufficient weight of evidence to counter powerful dominant discourses–psychiatry in this instance–and by reducing pressure to do this on individuals and their necessarily “single stories” [52]. A review of the characterisation of recovery narratives to date may make un-noticed assumptions more visible. This may enable a wider range of disparate narratives to be expressed. Considered collectively, it may become possible to identify potentially fruitful points of intervention in supporting recovery. A review may also highlight hitherto overlooked narratives in both research and practice contexts.

Taylor and colleagues [53] suggest that people recovering from mental health distress or trauma may find it helpful, in re-storying their experiences, to use such resources as the ‘Write to Recovery’ courses offered by the Scottish Recovery Network, or the ‘Telling Your Story’ courses run in Recovery Colleges around the world. Many organisations have also developed guidelines for telling stories, for example the U.S. Substance Abuse and Mental Health Services Administration [54]. An exploration of the characteristics of recovery narratives to date may help to inform the content or structure of such guidelines and courses. While emphasising that “other types of narrative can and should be proposed” [55], they may enable participants to make more meaningful sense of their experiences on the basis of “an increased stock of available narratives” ([56] p.285).

No systematic review of publications focusing on the characteristics of recovery narratives has previously been undertaken. Considered collectively, a greater diversity of narrative templates begins to emerge. A systematic review could provide a comprehensive overview of the multiple characteristics of recovery narratives which have, to date, been identified by researchers, while serving to highlight some gaps.

The review question was ‘how have mental health recovery narratives been characterised in academic literature?’ The aims were (a) to review published documents presenting typologies or characteristics of mental health recovery narratives and (b) to use a modified narrative synthesis to develop a conceptual framework for the characterisation of mental health recovery narratives.

## Methods

### Design

A systematic review of the literature was carried out following PRISMA guidance [57]. Studies meeting inclusion criteria and published in academic journals were assessed for methodological quality. A three-stage narrative synthesis of findings was undertaken using a modified version of Popay and colleagues’ guidance [58], to produce a conceptual framework of the characteristics of recovery narratives.

The review was conducted as part of the Narrative Experiences Online (NEON) Programme, and the systematic review protocol was registered with PROSPERO (presented in the supplementary material as S1 File).

### Eligibility criteria

The review was of studies investigating the characteristics of mental health recovery narratives. Mental health recovery narratives were defined, drawing on studies by Hall [46] and Thornhill and colleagues [48], as first-person lived experience accounts of recovery from mental health problems, which refer to events or actions over a period of time, and which include elements of both adversity/struggle and of self-defined strengths/successes/survival.

Inclusion criteria were:

- Presents or substantially advances an original framework of characteristics of mental health recovery narratives (including recovery from trauma and from childhood maltreatment)
- Based on empirical data.

Exclusion criteria were:

- Presents themes arising from narrative data without discussion of the characteristics of narratives themselves
- Not primarily or partially about mental health, e.g. recovery from chronic pain, physical illness or drug/alcohol addiction
- Based on third-person accounts, e.g. stories told by family, friends, carers, professionals
- Full text not available in English.

### Search strategy

A scoping search was undertaken and 12 publications were found. These informed the definition of mental health recovery narratives adopted by the current review (see above) and provided an overview of the nature of studies characterising mental health recovery narratives. The scoping search also informed the search strategy and confirmed that no systematic review had already been carried out in this area. Six search strategies were then selected to identify relevant publications.

The electronic database search strategy was developed and piloted in consultation with two research librarians with expertise in systematic reviews. Databases were selected by conducting preliminary searches to gauge relevance of results and degree of overlap with other databases; by including those indexing the journals of key publications found in the scoping search; and by expert consultation. Due to the cross-disciplinary nature of narrative research, databases from health sciences, social sciences and the arts and humanities were searched.

Fourteen bibliographic databases were searched from inception to 27^th^ July 2018: Applied and Complementary Medicine Database (AMED) accessed via OVID; Applied Social Science Index and Abstracts (ASSIA); Association for Computing Machinery Digital Library (ACM); Cumulative Index of Nursing and Applied Health Literature (CINAHL) via EBESCO; EMBASE; JSTOR; Linguistics and Language Behavior Abstracts (LLBA); Modern Languages Association International Bibliography (MLA) and Published International Literature on Traumatic Stress (PILOTS) Database both via ProQuest; MEDLINE; PsycINFO; Scopus via Elsevier; Arts and Humanities Citation Index and Social Science Citation Index, both via Web of Science.

A combination of subject heading and keyword searches was trialled to ensure sufficient specificity was maintained, while maximising the sensitivity of the search. MEDLINE was selected as the pilot database, as one of two databases which abstract the largest number of healthcare journals in the world [58]. The following search terms were used, identified from the title or abstract of papers:

1. Mental Disorders/
2. Behavior/
3. Psychological Phenomena/
4. Mental Health/
5. (Mental* or psych* or mad or madness or trauma* or distress* or ‘lived experience’).ti,ab.
6. 1 or 2 or 3 or 4 or 5
7. Mental Health Recovery/
8. Psychiatric Rehabilitation/
9. Resilience, Psychological/
10. Hope/
11. Quality of life/
12. (Recover* or transform* or resilien* or surviv* or thriv* or endur* or rebuild* or hope* or conquer* or reclaim*).ti,ab.
13. 7 or 8 or 9 or 10 or 11 or 12
14. Personal Narratives/
15. Narration/
16. Narrative therapy/
17. (narrat* or story or stories or storytelling or telling or tale* or restory* or counter-narrative* or disnarrat* or memoir* or testimon* or biograph* or autobiograph* or auto-biograph* or autoethnograph* or auto-ethnograph* or photovoice).ti,ab.
18. 14 or 15 or 16
19. (typol* or classif* or genre* or theme* or structur* or categor* or framework* or dimension* or format*).ti,ab.
20. 6 and 13 and 18 and 19.

Keyword and, where applicable, subject heading searches were subsequently tailored to each database.

The tables of contents of five journals were hand-searched from 1^st^ January 2000–27^th^ July 2018: BMJ Medical Humanities; International Journal of Narrative Therapy and Community Work; Journal of Medical Humanities; Anthropology and Medicine; Qualitative Health Research.

Journals were selected by expert consultation and by including those featuring two or more included papers from the original electronic data search. The start date was selected as the year before two prominent early papers on recovery narratives identified in the scoping search were published [44, 47].

Grey literature searches were conducted using Ethos, BASE and OpenGrey. Conference searches were undertaken using programmes available online from two recovery-oriented conferences: Refocus on Recovery (http://www.researchintorecovery.com/RoR-conference-archive) and ENMESH (http://www.enmesh.eu/Enmesh_Conferences.html).

Web-based searches were conducted using Google Scholar, ResearchGate and Academia.edu, and by searching the recovery-oriented websites Scottish Recovery Network (https://www.scottishrecovery.net) and Boston University Repository of Recovery Resources (https://cpr.bu.edu/resources/recovery-repository). Due to the large number of results found on Google Scholar (n = >644,000) only the first ten pages of results were searched.

A panel of 12 experts with expertise in mental health, design research, qualitative and narrative research was consulted for additional studies meeting the inclusion criteria.

Backward citation tracking was conducted by hand-searching the reference lists of all included papers. Forward citation tracking of papers citing included studies was conducted using Scopus (n = 832 results) and Google Scholar (n = 2082).

### Screening and eligibility assessment

Papers identified by the search were uploaded to Endnote, and duplicates removed. Titles were screened for relevance against the inclusion criteria by the lead researcher (JLB), with a randomly-selected sample of 10% double-screened for inclusion by a second researcher (SRE) to establish a pre-defined adequate concordance of 90% or above.

Potentially relevant abstracts were subsequently screened by the lead researcher (JLB). A sample of 20% of these abstracts was double-screened for inclusion by a second researcher (SRE). Full text was obtained for potentially relevant papers and eligibility decided by the lead researcher, with reasons for those excluded at full text retrieval stage documented in a PRISMA flow diagram.

### Data extraction and quality assessment

A data abstraction table was designed, and is presented in the supplementary material (S1 Table).

There is little consensus regarding the most appropriate way of evaluating qualitative evidence within systematic reviews [59]. However, in accordance with best-practice recommendations [60] the current study included a structured critical appraisal stage. The aim was not to exclude papers based on quality but to inform a subgroup analysis of those assessed as of moderate or high quality, in order to investigate any potential differences in findings or emphasis.

All studies published in academic journals were assessed for quality by two researchers (JLB and SRE). Qualitative studies were assessed using the Critical Appraisal Skills Programme qualitative checklist (CASP 2017), using thresholds modified from Butler and colleagues [59]. The Mixed Methods Appraisal Tool [61] was used to assess mixed methods studies. Other forms of publication (doctoral theses, government reports and books or book chapters) were excluded from the quality assessment.

### Data analysis

A three-stage narrative synthesis approach was used, modified from guidance for the conduct of narrative synthesis within systematic reviews [58]. Principles followed in the development of the synthesis were:

- to preserve study authors’ terminology in naming dimensions and types as much as possible, while maintaining clarity and avoiding potentially non-inclusive language. Authors’ original terms are preserved in the tables presenting sources of information for the synthesis.
- to avoid over-combining so as not to risk collapsing discrete concepts which might remain useful separately

A preliminary synthesis of studies using qualitative or mixed methods was developed by the lead researcher (JLB). Data were tabulated and analysed thematically to identify potential conceptual overlaps and/or similarities of language used to describe differing phenomena. Emerging dimensions fitted a framework commonly used in narrative and literary theory which considers narratives at three levels of form, structure and content (see for example [62]). These were adapted as superordinate categories.

Quantitative data, such as length of narratives or linguistic categories within narratives, did not form part of the narrative synthesis and are presented in summary form in the supplementary material (S2 Table):.

Relationships within and across studies were examined by the lead researcher (JLB), and subgroups of interest were identified. Publications within these subgroups were thematically analysed separately. Emergent themes were compared with the preliminary synthesis in order to identify areas of differing emphases and to assess robustness.

The robustness of the preliminary synthesis was assessed using the following methods: subgroup analysis of studies rated as moderate and high quality; subgroup analysis of studies where narrator(s) were involved in the analysis process (ranging from respondent validation to co-authorship); consultation with members of the NEON study Lived Experience Advisory Panel (LEAP) including people who have published their own mental health recovery narratives; consultation with an academic panel; and ongoing discussion and critical reflection by the research team. The synthesis was modified in response to findings; for example academic panel consultation strengthened internal coherence and LEAP consultation resulted in improved clarity of language used in the presentation of the synthesis and the definitions of dimensions and types.

## Results

### Results of literature search

**Fig 1:**
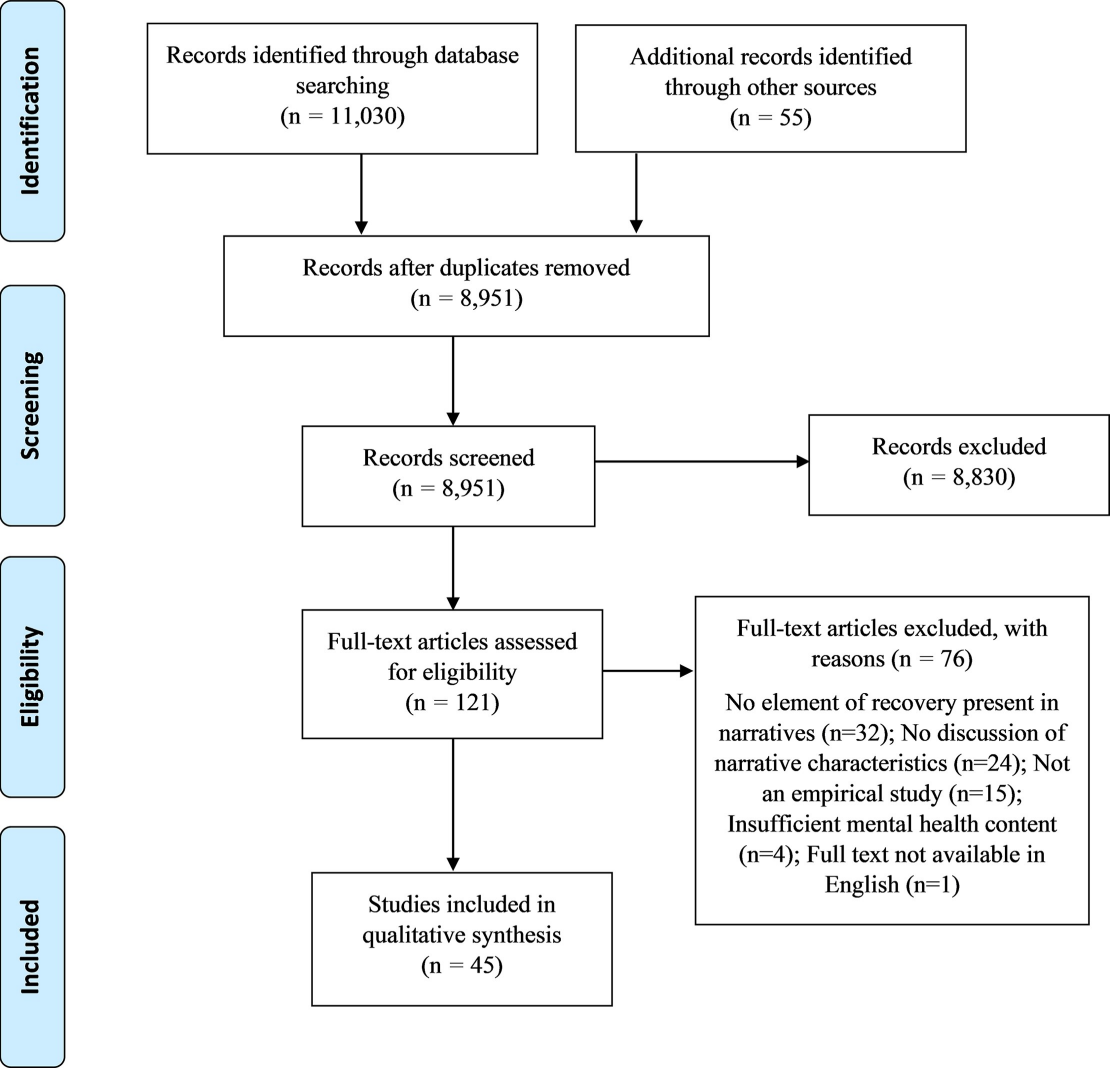
PRISMA flow diagram.

Forty-five publications were included in the review. Characteristics of included publications are presented in the supplementary material (S1 Table), and the flow diagram is presented as Fig 1 below:

### Included publications

The 45 publications comprise qualitative (n = 41) and mixed methods studies (n = 4). Studies were conducted by research teams based in 11 countries: the UK (n = 16), the USA (n = 16), New Zealand (n = 3), Australia (n = 2), Canada (n = 2), one each from Chile, Germany, Greece and Israel, and two from multinational teams (Australia/Netherlands/ India/USA and Australia, New Zealand and the UK). Publication was between 1997 and 2018. Fifteen of the 45 were published between 2006 and 2008.

Thirty-one publications explicitly use the term ‘recovery narrative’ or close variants to describe their source data. Variants include “stories of healing”, “redemption narratives” and “narrative re-storying”. This group includes a paper which contains, to the current authors’ knowledge, the earliest naming of ‘recovery narratives’ as a phenomenon distinct from ‘illness narratives’ [63]. Fourteen publications do not use ‘recovery’ or similar terminology to describe their source narratives; however, it was clear from either the methodology or results sections that elements of recovery were present within the narratives. Five of these 14 papers describe source data neutrally (e.g. personal narratives, life history narratives, user narratives or biographical narratives); three name them as narratives both of illness/trauma and recovery; three are named by the central activity under investigation (sport, football and activism narratives) and three name them solely as illness narratives or close variants (emotional distress narratives and anorexic experience narratives).

### Quality assessment scores

The 29 studies published in academic journals were assessed. Of the 25 qualitative studies, two (8%) were evaluated as high-quality, 10 (40%) as moderate-quality and 13 (52%) as low-quality. Of the four mixed-methods studies, two (50%) were evaluated as high quality, one (25%) as moderate and one (25%) as low quality. Sixteen studies were excluded from quality assessment, comprising doctoral theses (n = 11), reports (n = 3) and books or book chapters (n = 2).

### Participants

The 45 included publications analysed 629 first-person lived experience accounts. Gender of narrators was 59% (n = 370) female, 34% (n = 215) male and 7% (n = 44) not stated. Ages ranged from 8 to 79 years old, with 43 (96%) of publications only including adult (18 years or older) narrators. 40% of narrators were identified as white and 17% as BAME, while the ethnic identity of 43% of narrators could not be identified, as 26 of the 45 publications (58%) did not provide breakdowns of ethnicity. Narrators had experience of conditions or circumstances from across the spectrum of mental ill health. Those named (either by the research team or self-identified by narrators) were: anorexia, anxiety, bipolar disorder, borderline personality disorder, bulimia, childhood maltreatment, co-existing mental health and substance use issues, depression, dissociative identity disorder, eating disorders, mania, manic depression, nervous breakdown, personality disorders, psychosis, PTSD, schizophrenia, social anxiety, survival of sexual abuse and voice-hearing.

### Narrative synthesis

Nine dimensions were derived from thematic analysis of included publications, with each dimension containing a number of types. Types are not presented as discrete; some included authors state that more than one may be present within a narrative. The final synthesis is presented as Table 1 below:

**Table 1:** Characteristics of mental health recovery narratives.

SUPERORDINATE CATEGORY	NO.	DIMENSION	TYPES			
**Form**	**1.**	**Genre**	Escape	Enlightenment	Endeavour	Endurance
	**2.**	**Positioning**	Recovery within the system	Recovery despite the system	Recovery outside of the system	-
	**3.**	**Emotional tone**	Challenging	Disenfranchised	Reflective	Buoyant
			Shaken	Tragic	-	-
	**4.**	**Relationship with recovery**	Recovered	Living well	Making progress	Surviving day-to-day
**Structure**	**5.**	**Trajectory**	Upward spiral	Up and down	Horizontal	Interrupted
	**6.**	**Use of turning points**	Restorying	Change for the better	Change for the better or worse	-
	**7.**	**Narrative sequence**	Experience of distress/ trauma	Turning point	Experience of recovery	-
**Content**	**8.**	**Protagonists**	Personal level	Socio-cultural level	Systemic level	-
	**9.**	**Use of metaphor**	Distress metaphors	Recovery metaphors	-	-

### Superordinate category: Narrative form

Four dimensions related to narrative form were identified: Genre, Positioning, Tone and Relationship with Recovery. The central question common to all four dimensions is ‘what kind of story is this?’

#### Dimension 1: Genre

Twelve publications identified different genres of mental health recovery narrative, also using the following synonyms for genre: narrative type, form, plot and theme. Four types were synthesised from the 20 genres presented, using terms adapted from Thornhill and colleagues’ study [48]. These are shown in Table 2:

**Table 2:** Four ‘Genre’ types synthesised from included publications (n = 12).

SOURCE:	REF.	GENRE TYPES:			
		Escape	Endurance	Endeavour	Enlightenment
**6**	**[64]**	Escape	_	Endurance/ acceptance	Exploration/ discovery
**11**	**[65]**	Survival	Salvage	_	Growth
**18**	**[66]**	-	Turning away/protective hibernation	-	Turning towards/ empowerment
**21**	**[63]**	-	-	-	Spiritual quest
**30** **32** **36**	**[67]** **[68]** **[69]**	-	-	-	Quest
**33**	**[70]**	-	-	-	Humanistic quest
**35**	**[71]**	-	-	Normalising	Conversion/growth
**41**	**[72]**	-	Recovery in the midst of chaos	Recovery as restitution	Recovery as quest
**42**	**[49]**	-	-	-	Redemption
**44**	**[48]**	Escape	Endurance	-	Enlightenment

Source: ID number in Data abstraction table. Ref: citation number.

All 12 publications sought to identify genres in order to examine constructions of meaning by narrators. Nine publications (75%) cited Frank’s genres of restitution, chaos and quest illness narratives [25] as either influential in identifying further recovery narrative types, or as being directly applicable to their own research data.

The ‘Escape’ type comprises narratives of escape from and resistance to abuse, threat, stigma and persecution. Images of entrapment and/or of a fight for survival may be used [48]. Escape can be from oppressive beliefs, systems, services or treatments. It may also refer to the narrator’s escape from a negative internalized identity, as a result of maltreatment or stigma [65].

The ‘Endurance’ type comprises narratives of loss, trauma, difficult circumstances and/or seemingly insurmountable odds. They may employ images of weathering storms or battening down the hatches to conserve energy [66]. They may contain haunting or chaotic elements, or describe being in the midst of traumatic events [71]. Success may be expressed in terms of having survived, or kept going—the narrator’s priority may be salvaging, over restoring or transforming themselves [65].

The ‘Endeavour’ type comprises narratives incorporating positive aspects, coping strategies and/or plans, and an acceptance of difficulties as an ongoing factor of recovery. Narrators may feel they are active agents of change [64], or they may focus on doing things or keeping busy [71]. Their priority may be managing or restoring order, rather than transforming themselves [72].

The ‘Enlightenment’ type comprises narratives of transformation. The narrator views the illness/trauma as ultimately positive, as new perspectives have been gained from it. They may describe recovery as a journey of exploration or discovery [64], leading to empowerment and/or self-actualisation [66]. The narratives may contain aspects of redemption [49]; of having been saved by something greater than themselves, either by spiritual [63] or humanistic [70] means.

#### Dimension 2: Positioning in relation to clinical model

Eleven publications identified ways in which mental health recovery narratives can be positioned in relation to the clinical model of mental health. The following synonyms for positioning were also used: major theme/plot, typologies of narratives genre, and narrative types. Unlike those in Genre, these publications specifically foreground social and political considerations of the mental health system in the identification of narrative types. The ‘mental health system’ is defined for the purposes of this paper as being the dominant clinical mental health provision of the country involved. Three types were synthesised from the 13 positions presented, using terms identified by the current research team. These are shown in Table 3:

**Table 3:** Three ‘Positioning’ type synthesised from included publications (n = 11).

SOURCE:	REF.	POSITIONING TYPES:		
		Recovery within the system	Recovery despite the system	Recovery outside of the system
**1**	**[73]**	Traditional narratives	Counter-narratives	Alternative or “good-life” narratives
**3**	**[50]**			
**4**	**[74]**			
**32**	**[68]**			
**2**	**[75]**	Psychiatric empowerment narratives	Psychiatric oppression narratives	Healing narratives
**12**	**[76]**	-	-	Adventure stories
**14**	**[77]**	-	-	Narratives of activism
**15**	**[78]**	-	-	Action/achievement/ relationship narratives
**16**	**[55]**	Restitution narratives	Counter-narratives	-
**23**	**[56]**	-	Transgressive/resistance narratives	-
**31**	**[79]**	-	Divergent stories	-

Source: ID number in Data abstraction table. Ref: citation number.

‘Recovery within the system’ comprises narratives incorporating positive experiences of clinical mental health services. Diagnosis may be experienced as empowering, and treatment, services and/or relationships with practitioners as enabling, positive or a salvation.

‘Recovery despite the system’ comprises narratives of protest, in opposition to the clinical model of mental illness and/or mental health services and systems. They may incorporate experiences of maltreatment by mental health services. They may seek to recover the narrator’s own voice, sense of agency and purpose [50], and may also seek to raise social awareness and challenge psychiatric authority [75].

‘Recovery outside of the system’ comprises narratives in which clinical mental health services do not feature, or feature only minimally. They may not engage with psychiatric definitions or psychological concepts of personal growth; presenting experiences of living a “good life” beyond a focus on individual factors [74]. They may incorporate social, political, spiritual, and economic factors, often with a focus on specific areas such as activism [77], adventure [76] or relationships [78]. They may contain elements of having a greater purpose–“helping others in the same boat”–and/or a changed understanding of what is most important in life [75].

#### Dimension 3: Emotional tone

Three publications identified different emotional tones present within mental health recovery narratives, with one using the term “self-positions” [80]. Six types were synthesised from the 22 tones presented, using terms identified by the current research team. These are shown in Table 4:

**Table 4:** Six ‘Emotional Tone’ types synthesised from included publications (n = 3).

SOURCE:	REF.	EMOTIONAL TONE TYPES:					
		Critical	Disenfranchised	Reflective	Buoyant	Shaken	Tragic
**24**	**[80]**	Defiant	Subordinate	Reflective-conciliatory	-	-	-
**30**	**[67]**	Challenging, critical, sarcastic, argumentative	Passive, anxious	Grateful	Confident, hopeful	-	Tragic
**44**	**[48]**	Angry, protesting, educating	Resigned, disenfranchised, monotone	Educating, thoughtful	-	Disbelieving, shocked	-

Source: ID number in Data abstraction table. Ref: citation number.

#### Dimension 4: Relationship with recovery

Nine publications identified different ways of relating to the concept of recovery within mental health recovery narratives. The following synonyms were also used: recovery talk, narrative positions or types, framings of recovery, narrative motifs, core narratives and narrative genres. Four types were synthesised from the 15 presented, using terms adapted from Barnett and Lapsley’s typology [81]. These are shown in Table 5:

**Table 5:** Four ‘Relationship with Recovery’ types synthesised from included publications (n = 9).

SOURCE:	REF.	RELATIONSHIP WITH RECOVERY TYPES:			
		Recovered	Living well	Making progress	Surviving day to day
**9**	**[81]**	-	Living well	Moving forward	Surviving day to day
**10**	**[82]**	-	Healing	Incipient healing	-
**17**	**[83]**	Well	-	-	Getting by
**19**	**[84]**	Resolute narratives	Reconciliation narratives	-	-
**25**	**[46]**	-	Struggling successfully	-	Struggling daily
**28**	**[85]**	-	Ongoing recovery	-	-
**37**	**[47]**	-	Ongoing journey	-	-
**38** **39**	**[86]** **[87]**	Full recovery	-	-	Struggling recovery

Source: ID number in Data abstraction table. Ref: citation number.

The ‘Recovered’ type comprises narratives presenting recovery as an outcome which has been achieved. Narrators see the illness or distress as being in the past [83]. There may be a clear split between past and present selves [87].

The ‘Living well’ type comprises narratives presenting recovery as a process within which the narrator is well-established. Narrators are living well in the presence or absence of mental illness or distress [81], and see any continuing difficulties as things which they can overcome [82].

The ‘Making progress’ type comprises narratives presenting recovery as a process in which they are seeing some progress. Narrators present confidence in their ability to cope despite feeling relatively close to the disruptions of mental distress or trauma [81].

The ‘Surviving day to day’ type comprises narratives presenting recovery as a journey on which the narrator is tentatively engaging. The narrator may be in a new, difficult or dangerous situation [46] where it may be difficult to realise their hopes [81], but they still express their experiences in a recovery context [83].

### Superordinate category: Narrative structure

Three dimensions related to narrative structure were identified: Narrative Trajectory, Use of Turning Points and Narrative Sequence. The central question common to all three is ‘what shape does this story take?’

#### Dimension 5: Trajectory

Seven publications identified different trajectories present within mental health recovery narratives, also using the following descriptions: types of emotional distress, narrative shapes, plots and structures. These may describe the shape of whole narratives or of sequences within narratives. Four types were synthesised from the 14 presented, shown in Table 6:

**Table 6:** Four ‘Narrative Trajectory’ types synthesised from included publications (n = 7).

SOURCE:	REF.	TRAJECTORY TYPES:			
		Upward spiral	Up and down	Horizontal	Interrupted
**2**	**[75]**	Revelation/purposeful suffering	-	Continuity	Traumatic interruption
**7**	**[88]**	Spiralling towards health	Progressive and regressive courses of action	-	-
**13**	**[89]**	-	Progression with downs as well as ups	-	-
**27**	**[90]**	Expecting	-	Accepting	-
**36**	**[69]**	Quest/progressive narratives	-	Restitution/stability narratives	-
**42**	**[49]**	Steady upward progression	Roller-coaster	Struggling/stagnating	-
**45**	**[91]**	-	Journey which may move towards health or towards illness	-	-

Source: ID number in Data abstraction table. Ref: citation number.

The ‘Upward spiral’ type comprises narratives describing a journey with an overall ascending progression toward recovery. They may be described as narratives of revelation or purposeful suffering [75], or evolution from darkness to light towards a better future [88], or of overall improvement. Setbacks might occur, but they are defined as solvable problems [49].

The ‘Up and down’ type comprises narratives describing a non-linear journey which challenges the progressive trajectory of spiralling ever forward towards health [88]. They contain continuing upturns towards health/wellbeing and downturns towards illness/struggle, which may be experienced as dramatic, “roller-coaster” narratives [49] or narratives with “downs as well as ups” [89].

The ‘Horizontal’ type comprises narratives without significant upturns or downturns. The narrator may feel that they are stagnating [49], or taking one day at a time [69].

The ‘Interrupted’ type comprises narratives describing a journey interrupted by an unexpected crisis or difficulty, after which the narrator’s life has returned to its prior state [75].

#### Dimension 6: Use of turning points

Eleven publications identified turning points as being a characteristic of mental health recovery narratives, but defined ‘turning points’ in three different ways. Three types were synthesized, shown in Table 7:

**Table 7:** Three ‘Turning Point’ types synthesised from included publications (n = 11).

SOURCE:	REF.		TURNING POINT TYPES:		
			Re-storying	Change for the better	Change for the better or worse
**7**	**[88]**		Narrators resist dominant narrative and take ownership of own stories	**-**	**-**
**8**	**[92]**		-	Large shifts/changes leading to improvement	-
**10**	**[82]**		-	-	Critical life events, either positive or negative, which lead to changes in one’s lifespan.
**11**	**[65]**		-	-	Significant transitions or disruptions to a trajectory or turns in narrative accounts
**13**	**[89]**		-	Point at which the opportunity to begin a recovery journey can present itself	-
**25**	**[46]**		-	-	A point in the narrative trajectory, after which immediately subsequent events may be negative or positive
**26**	**[93]**		Points which open possibilities to re-story experiences and arrive at new understandings	-	-
**29**	**[94]**		-	Dramatic moments [leading to positive change]	-
**33**	**[70]**		-	The point of realizing others couldn’t help, or the catalyst for [positive] change	-
**35**	**[71]**		Transition points from dominant/stigmatising narrative to personal/ positive stories	-	-
**42**	**[49]**	-		-	Points in the narrative followed by "redemption sequences" or "contamination sequences"

Source: ID number in Data abstraction table. Ref: citation number.

The ‘Re-storying’ type comprises narratives within which turning points are considered as moments in which a narrator gains a new understanding of their experiences [93]. It may be the point at which a narrator resists being defined by a dominant discourse and takes over authorship of their own story [88], or the transition point from a stigmatising narrative to a positive one [71].

The ‘Change for the better’ type comprises narratives within which turning points are considered as moments of transition, followed by sequences leading to improvement [92] or positive change [94]. They may be positive events in themselves, such as a moment of self-acceptance or intervention from others [89], or difficult moments which prove to be a catalyst for positive change, such as realising that others couldn’t help them [70].

The ‘Change for the better or worse’ type comprises narratives within which turning points are considered as critical life events [82] or significant transitions or disruptions in the narrative [65], followed by “redemption” or “contamination” sequences [49] where events may be negative or positive [46].

#### Dimension 7: Narrative sequence

Eight publications identified different sequences within mental health recovery narratives, also using the following terms: stages, narrative shape, typologies and structure. Eight types were synthesised from the 37 sequences described. These are shown in Table 8:

**Table 8:** Eight ‘Narrative Sequence’ types synthesised from included publications (n = 8).

SOURCE:	REF.	NARRATIVE SEQUENCE TYPES:							
		Life before distress/ trauma	Problems begin	Problems worsen	Impact of illness/trauma	Glimpses of recovery	Turning point	Roads to recovery	Life afterwards
**9**	**[81]**	Life before the mental health crisis	Going downhill and seeking help	The mental health crisis	-	**-**	Contact with mental health services	Recovery	Reflections
**12**	**[76]**	Sporting histories	Problem stories	-	-	-	Getting involved in sport	Personal benefits, community and connection	Staying involved
**13**	**[89]**	Life before illness	-	Life during illness	-	Glimpses of recovery	Critical incident leading to change in perception or realisation recovery is possible	Recovery	Hope for a better future
**21**	**[63]**	-	Non-recovery	-	-	-	-	Recovering period	Recovered with ongoing transformation
**23**	**[56]**	-	Narrative disruption				Narrative repair	Narrative re-storying	-
**29**	**[94]**	Origins	Onset	Experience of mental illness	Consequences of illness	Glimpses of recovery	Turning point	The road to recovery	Life afterwards
**34**	**[14]**	-	-	-	-	-	Expression of [potentially] previously hidden suffering	Logical organising of experience allowing for new perspective	Inclusion of hopeful and/or triumphant elements in order to inspire others
**40**	**[95]**	-	Traumatic past	-	-	-	An episode of change	Ongoing recovery phase	-

Source: ID number in Data abstraction table. Ref: citation number.

### Superordinate category: Narrative content

Two dimensions related to narrative content were identified: Protagonists and Use of Metaphor. The central question common to them both is ‘what resources have been deployed in the telling of this story?’

#### Dimension 8: Protagonists

Four publications identified different protagonists within mental health recovery narrative, also using the following synonyms: narrators, biographical types, archetypal protagonists, major players and heroes/supporting cast. Three types were synthesised from the 15 protagonists presented, shown in Table 9:

**Table 9:** Three ‘Protagonist’ types synthesised from included publications (n = 4).

SOURCE:	REF.	PROTAGONIST TYPES:		
		Personal level	Socio-cultural level	Systemic level
**6**	**[64]**	The strong conqueror The scarred survivor The enlightened explorer	-	-
**20**	**[96]**	The self/narrator The bulimia/illness		The environment/outer worlds
**22**	**[97]**	Users/consumer Survivors	-	-
**36**	**[69]**	The hero The illness Medication	Mental health workers/agencies Family Friends	Community

Source: ID number in Data abstraction table. Ref: citation number.

‘Personal factors’ are the micro-level or inter/intra-personal factors within a mental health recovery narrative. Most commonly the narrator him or herself, who may be characterised [64] or positioned [97] in various ways, these may also be helping or hindering persons or factors such as medication, form of treatment or mental health professional [69]. The illness or traumatic situation itself may function as an intra-personal protagonist in terms of being a driving force within the narrative [96].

‘Socio-cultural factors’ are the meso-level factors within a mental health recovery narrative, including family and friendship dynamics, groups or local organisations, mental health staff and services. These may be “supporters or villains”, exerting positive or negative effects on the narrative [69].

‘Systemic factors’ are macro-level factors within a mental health recovery narrative, named in included publications as the wider community [68], and the environment or outer worlds [96], but also potentially including legal, healthcare, policy, political and international factors which affect the narrative either positively or negatively.

#### Dimension 9: Use of metaphor

Three publications focused on the use of metaphor within mental health recovery narrative. Two types were synthesised from the six presented. These are shown in Table 10:

**Table 10:** Two ‘Metaphor’ types synthesised from included publications (n = 3).

SOURCE:	REF.	METAPHOR TYPES:	
		Distress metaphors	Recovery metaphors
**17**	**[83]**	Ill metaphors	Healthy metaphors
**20**	**[95]**	Illness metaphors	Recovery metaphors
**43**	**[98]**	Distress metaphors	Recovery metaphors

Source: ID number in Data abstraction table. Ref: citation number.

‘Distress metaphors’ may depict a deep descent of the self, a “spiralling out of control” [83]. They may convey a sense of disconnection and alienation, or of chaos, lack of control, loneliness or suffering [96]. They may be focused on past or current distress or an imagined future return to the experience of distress [98].

‘Recovery metaphors’ may depict health as the main road to which one must return [83]. They may convey a sense of connection, bonding and integration, a regaining of control of life, partnership with others, or victory in the fight against illness [96]. They may be focused on past, present or hoped-for future experiences of recovery [98].

### Subgroup analyses

Four subgroup analyses were undertaken of papers published in academic journals. Analysis of moderate and high-quality papers (n = 15, 52%) found that the following items from the conceptual framework were not present: two dimensions (Protagonists and Use of Metaphor); one type of Genre (‘Endeavour’); two types of Emotional Tone (‘Buoyant’ and ‘Tragic’), and a type within Relationship with Recovery (‘Recovered’). Within the Narrative Sequence dimension three types were found (‘Experience of psychological distress/trauma’, ‘Turning point’ and ‘Experience of recovery’).

Analysis of papers with narrator involvement at analysis stage (n = 11, 24%) found that eight of the nine dimensions and all corresponding types were present. The Emotional Tone dimension and its types were not found in these papers. Within the Narrative Sequence dimension eight more detailed types were found (‘Life before illness/trauma’; ‘Problems begin’; ‘Problems worsen’; ‘Impact of illness/trauma’; ‘Glimpses of recovery’; ‘Turning point’; ‘Roads to recovery’; and ‘Life afterwards’).

Analysis of papers focusing exclusively on narratives of psychosis (n = 12) found no significant differences of emphases when compared with papers focusing on other conditions.

Subgroup analysis found that all papers focusing on trauma (n = 6) focused on dimensions within the superordinate category of Narrative Structure, namely Trajectory, Use of Turning Points and Narrative Sequence. All six discussed Use of Turning Points. One also included discussion of Genre [48].

## Discussion

This review has identified the existence of a sizeable body of qualitative and mixed-methods literature describing the multidimensional ways in which mental health recovery narratives have been characterised. Forty-five publications were identified by the systematic review search. The literature was multidisciplinary and published in a wide range of journals, spanning 21 years of research. The 45 papers represent analysis of a total of 629 first-person lived experience accounts of psychological distress and recovery, from narrators representing many demographics. A key contribution of this review is to collate and synthesise these disparate narratives. This provides an accessible resource for practitioners, researchers and others interested in the distinctly heterogeneous ways in which people both recover and narrate their recovery from psychological distress and/or trauma. It will also inform a future trial of the impact of accessing recovery narratives online on the quality of life of people experiencing psychosis (ISRCTN11152837).

The narrative synthesis then moves beyond this work by providing a more comprehensive framework for characterising narratives than any single model can offer. The synthesis found that mental health recovery narratives can be characterised under three superordinate categories of narrative form, structure and content, and nine dimensions: Genre, Positioning, Emotional Tone, Relationship with Recovery, Trajectory, Use of Turning Points, Narrative Sequence, Protagonists and Use of Metaphor. This extends previous work such as that of Frank [23, 24] and offers new directions for research and practice.

### Recovery narratives present diverse, fluid meaning-making processes

A key finding of this review is that none of the included publications claim a ‘right way’ to narrate recovery or to characterise mental health recovery narratives. Care is taken by authors of included publications to stipulate that narrators speak from a wide range of circumstances, and that no type of recovery narrative should be pathologised or, by implication, valorised, in relation to others [66].

Narrators do not present single or static types of narratives. As with “small stories” or exchanges in everyday life [99], narrators of recovery stories work with interpretive repertoires [100] which can be revised [101], allowing narrators to manage their positions to suit different purposes, audiences and contexts. Narratives may change over time, as for example in Howard’s 2006 study of exiting recovery identities [90].

Correspondingly, the current conceptual framework is not presented as a definitive or exhaustive list of types; but as a “network or ‘plane’ of linked concepts that together provide a comprehensive understanding of a phenomenon” ([102] p.51). Following the example of Smith and Sparkes ([18] p.2), the aim is not to suggest what a recovery narrative *is*, but what its possibilities are—what it *can be*. Narrators express their recovery through many types and shapes of story, drawing on a variety of resources to do so. This is appropriate for a recovery paradigm wherein meaning-making is a highly individual process [3]. It echoes Jacobson’s 2001 point recalled by Spector-Mersel and Knaifel, that recovery narratives “teach us to respect pluralism and difference, cautioning us from offering the one and only path towards a ‘proper’ recovery” ([10] p. 5).

### Recovery narratives are multidimensional

Mental health recovery narratives express individual meaning-making processes within recovery; they also highlight wider socio-economic and systemic influences. Narrators in the included publications populate their recovery narratives with supportive and hindering factors at individual, socio-cultural and systemic levels. This finding supports research presenting recovery as a multidimensional process, involving biomedical, psychological, social and socio-political resources and components [103]. It responds to concerns raised by Fisher and Lees [51] that recovery should not be reduced or erased by emphasis on narratives of, for example, individual progress towards economic independence.

### Recovery narratives are distinct from illness narratives

Recovery narrative analysis in the included publications is rooted in influential work within the medical humanities on illness narratives, notably by psychiatrist and anthropologist Arthur Kleinman [104] and medical sociologist Arthur Frank [24, 25]. However, the current synthesis finds that there are significant additions and differences of emphases which mark mental health recovery narratives as a distinct phenomenon from physical illness narratives. It challenges and extends Frank’s original typology of restitution, chaos and quest narratives [25] as follows:

#### Recovery narratives incorporate social, political and human rights factors

Recovery narratives have been characterised by genre, as have illness narratives, but add a new type, first identified by Thornhill and colleagues: narratives of “Escape” [48]. The presence of these narratives, characterised by escape from services or from treatments experienced as damaging, highlights the political factors involved in mental health recovery. It supports human rights-based approaches to mental health recovery [105], and approaches which de-emphasise individual recovery factors in favour of the role of transformation of systems and services [106].

Recovery narratives are additionally characterised by consideration of narrative positioning in relation to a clinical model of mental health. Positioning relates to the “social and emotional stances that individuals take vis á vis real or imagined others” ([107], p.171). Concern has been expressed that the original emancipatory role of narratives within mental health movements is at risk of being lost through their assimilation within services, with a focus on individual factors alone [51]. The ‘Recovery despite the system’ and ‘recovery outside of the system’ types of narrative address this by foregrounding the importance of inter-personal and social factors within recovery, such as positive relationships and participation in sports or arts activities, and of addressing systemic factors, such as through activism.

#### Recovery narratives include recovery outside of and within mental health services

Frank’s [25] restitution type of illness narrative foregrounds the dominant clinical model of illness. Similarly, the current review includes studies identifying narratives of ‘recovery within the system’, wherein narrators have experienced treatment, medication and/or relationships with mental health staff as positive factors in their recovery. However, within a professional culture which can tend to be disempowering and problem-focused [108], narratives of mental health recovery in any form can be seen as empowering counter-narratives to more dominant discourses. In this context, narratives of ‘Recovery within the system’ can be seen as radical in their own right, representing a challenge to the therapeutic pessimism which can exist around certain mental health conditions [109].

Mental health recovery narratives also challenge the clinical model more directly, providing evidence and mirroring research that recovery can happen without the intervention of services [110]. Narratives of ‘Recovery despite the system’ emerged from psychiatric survivor movements, and directly oppose dominant clinical discourses. Narratives of ‘Recovery outside of the system’ disengage altogether from services and treatment, and in this context the dominant clinical discourse is not relevant.

Narratives opposing or outside of the mental health system have played a crucial role in survivor activism, and ultimately in establishing recovery as an orientation within services. Given this historical context, narratives endorsing mental health treatments or services may be dismissed as conforming to the dominant clinical model, rather than expressing authentic experience–the narrator perhaps performing the role of the ‘good patient’. The potential for narrative conformity in particular groups has been researched, including Frank’s own exploration of Alcoholics Anonymous groups [24]. However, dismissing narratives of ‘Recovery within the system’, or any particular type of narrative, risks creating new “dominant recovery narratives” ([14], p.26) and dismissing the equally valid experiences of those for whom interaction with the mental health system has been positive. Diagnoses, for example, have been identified by some as useful resources in maintaining a positive self-concept [111].

#### Recovery narratives challenge diagnostic master narratives and narrative theory itself

Frank [25] defines his chaos narratives as lacking narrative order, with a central plot that life can never get better. This mirrors research which finds the narratives of people experiencing particular conditions, for example psychosis, as inherently lacking insight or coherence. Chaotic narratives are here equated with continuing illness, or seen as the essence of the illness itself (see for example [112]). The current review challenges this finding in several ways.

Firstly, the review includes narratives (synthesised as ‘Endurance’ narratives) which present recovery as being possible in the midst of considerable chaos (see for example [72]). Chaotic narratives may still be recovery narratives.

Secondly, subgroup analysis of studies focusing on narratives of psychosis found no difference in characteristics or emphasis when compared with narratives of other forms of psychological distress. Insofar as psychosis involves chaos, it is noteworthy that people experiencing psychosis narrate recovery with the same characteristic features and multi-dimensional meanings as other narratives. This finding challenges the stereotype of people experiencing psychosis as being incompetent or incapable of insight into their experiences. It supports moves to broaden the concept of ‘insight’ within psychosis services to incorporate the ‘narrative insight’ [113] or explanatory frameworks of those experiencing the psychosis. It has been noted that narrative insight is a different skill to promoting clinical insight, and may require new approaches which avoid privileging explanatory clinical models [114]. This review supports those interventions which take a joint, non-directive approach to exploring narratives between practitioner and service user, for example narrative therapy [40], or MERIT, a recently developed metacognitive approach [115]. The joint exploration of an individual’s narrative may provide mutual insight and understanding, offering a practitioner access to the meaning-making which is already present for an individual, and transforming for the practitioner that which may previously have been seen as ‘incoherent’, as well as supporting the individual to make sense of their experiences. Thirdly, and paradoxically, the implication that coherence is a prerequisite of meaningful narrative is in itself problematic. Although Frank asserts a moral and clinical imperative to honour chaotic tellings, he still describes chaos as “the pit of narrative wreckage” ([25], p. 110). Theorists have been criticised for imposing requirements of coherence on narrative [116], and the pre-occupation with linearity is contested by health humanities and other critical theories [34]. The current review supports this criticism. Such requirements of narrative were abandoned by literary theorists, writers and poets in favour of modernist and post-modernist approaches over one hundred years ago. Instead of being anti-narrative, the ‘Endurance’ type redefines narrative to include those with elements of “fragmentation, amorphousness, entropy, chaos, silence, senselessness” [33]. This allows the inclusion of narrators who do not story their recoveries as linear, and/or those whose experiences are not easily expressed within the limits of language.

#### Recovery narratives may help identify and generate post-traumatic growth

Frank’s quest narratives [25], synthesised here as ‘Enlightenment’ narratives, could be hypothesized with their emphasis on transformation and growth to be the most likely fit with recovery narratives. Indeed, this is the type most discussed by publications characterising by genre. ‘Enlightenment’ narratives represent the narrator’s re-storying of events to demonstrate growth which has occurred as a result of the original trauma or distress. Characteristics of enlightenment identified by included publications narratives parallel the five domains of post-traumatic growth (PTG) identified by Calhoun and Tedeschi [117], and it has been suggested that analysis of narrative accounts may be the most valid way of assessing for PTG [118]. Eliciting narratives is a retrospective method, which may have weaknesses in terms of correlating with actual psychological change [119]. However, retrospective measures have been found to correlate moderately to well-being outcomes [120], which Jayawickreme and Blackie note is worthy of further research in itself [121]. Further research on enlightenment narratives may contribute to understanding in this area, for example by investigating whether opportunities to undertake such re-storying may be a means of fostering, as well as evaluating, post-traumatic growth for individuals such as those living with psychosis [122].

### Recovery narratives can be non-linear

A core domain of recovery is its non-linearity [123]. This is reflected in the Trajectory dimension of the current synthesis, primarily by the ‘Up and down’ type which incorporates examples such as “lengthy, progressive and regressive” narratives, “roller-coaster” narratives, or “progression with downs as well as ups”. Anderson and Hiersteiner [88] provide an account in their study of recovery from childhood sexual abuse of being explicitly challenged by their participants on their assumption of inviolate progress. Even the ‘Upward spiral’ trajectory type reflects this, qualified as being “steady” upward progress with recognition of setbacks [88].

In contrast, the Narrative Sequence dimension comprises surprisingly linear progressions through eight sequences, from experiences of distress/trauma through a turning point to recovery. Following DiClemente and Prochaska’s lead with their transtheoretical model of change [124], it would be possible to adapt presentation of this theme to represent a more non-linear or spiraling progression, with narrators returning to earlier sequences or cycling through them. This adaptation is further validated by the ‘Change for the better or worse’ type of Turning Point, which does not assume straightforward progression, and links recovery narratives with research on lives in transition such as the “contamination” and “redemption” sequences of McAdams and Bowman [125]. Further research could be undertaken to investigate whether a non-linear or spiral model is a better fit for some narrators’ experiences of recovery.

### Narratives and narrators currently missing from the literature

An important contribution of the current synthesis is to highlight the kinds of recovery narratives, narrators or other factors missing from research to date, and some significant gaps were noted. One such gap was that only one of the included publications provided a clear definition of mental health recovery narratives [46].

All included publications except one focused on the narratives of individuals. The exception was Anderson and Hiersteiner’s study which used group interviews to construct a group narrative of recovery-survival of childhood sexual abuse [88]. Further research could be undertaken on the characteristics of collective narratives, which may more accurately represent the significance of socio-economic and systemic factors in psychological distress, highlighted within survivor activism, Mad Studies [126], and recent survivor-informed psychological frameworks [127].

All narratives in their original formats were either written or spoken, and none was accessed online. Two publications complemented the collection of written/spoken narratives by additionally undertaking art and music-based interventions [71, 91]. Work has been undertaken to capture non-text based recovery narratives via other media, for example using visual methods such as Photovoice [128], and future research could examine the characteristics of these narratives for similarities and differences. Narratives shared online are also likely to have different characteristics [126] and future research could explore the implications of this.

Some included publications focus on “exemplar” narratives or “seminal accounts”, for example [47, 48, 56, 79]. These foreground narrators who possess “the transcultural, intellectual, cultural and symbolic capital to tell their tales with considerable authority” ([56], p.281) and who commonly relate ‘enlightenment’ narratives. This may have been an important choice in the beginning of survivor activism, in terms of mobilizing survivor self-determination [56] and challenging dominant discourses. However, the resources and social capital available (or not) to a narrator influence the kinds of narratives they may be able to relate. De Jager and colleagues [66], in their study of narratives of hearing voices, note for example that narrators of their “empowerment” narratives were all members of Hearing Voices Network groups and also had access to supportive professionals, whereas narrators of “protective hibernation” narratives did not have access to such resources. Future research could focus on the recovery narratives of those who have not had access to significant resources or support, which may extend the current framework.

Finally, although the presence of socio-cultural and systemic-level factors in recovery were noted by two studies [69, 96], represented in the conceptual framework within the Protagonists dimension, there was little discussion within analysis of how multiple forms of structural oppression can intersect and be mutually reinforcing. Future research could take a narrative approach more informed by intersectionality theory, as recent narrative analyses within systematic reviews have chosen to do [129].

### Strengths and limitations of the review

The comprehensive search strategy is one strength of this review, reflecting multidisciplinary interest in narrative approaches. Another is the quality assessment process. In addition to standard critical assessment of methodological quality, a subgroup analysis was carried out on studies incorporating narrator validation or co-production, as a complementary measure of trustworthiness. This is in line with the principles of agency and giving voice to potentially marginalised groups which are central to both recovery and narrative paradigms [10]. It is noted that 24% (n = 11) of included studies involved narrators themselves at analysis level. Further research on recovery narratives could ensure greater co-production of findings.

The robustness of the synthesis is another strength. Subgroup analyses of studies of high methodological quality and of high trustworthiness validated the findings of the preliminary synthesis. Expert consultation was carried out with academic experts, which strengthened the internal coherence of the synthesis, and a Lived Experience Advisory Panel, which increased the trustworthiness and ecological validity of the synthesis.

A further strength of this review is that its first and second authors have dual identities as both researchers and people with lived experience of mental ill health and recovery. This informed in particular the principles adopted in the development of the narrative synthesis.

The review is limited by its search for publications available in English only. This led to included publications which in the main reflect criticism that research into recovery is monocultural [130]. It is likely to have resulted in a framework which may not adequately reflect the characteristics of recovery narratives and mental health distress worldwide, as recent similar work has found [131]. Further research could be conducted on the characteristics of non-English language recovery narratives, in order to broaden understanding of cross-cultural or global mental health recovery.

A second limitation is that the involvement of an external Lived Experience Advisory Panel was consultative. The emergence of models of collaborative data analysis [132] can support more meaningful involvement of external panels of people with lived experience as co-analysts than took place in this review.

### Implications for practice

#### A transdiagnostic approach

The reviewed publications include narratives across the range of conditions and experiences of psychological distress. Subgroup analysis of psychosis and of trauma studies found no important differences in their characterisation compared with those narrating other conditions. This resonates with research on transdiagnostic approaches [133], which reject mental health perspectives based on competing theoretical positions in favour of an inclusive approach to treatment or services, incorporating multiple perspectives [134].

#### Using the framework as a diversity tool

The framework provided by the current synthesis could form the basis for an inventory of recovery narrative characteristics. This could be used by those delivering narrative-based interventions as an evidence-based template, ensuring diversity in two ways.

Firstly, such an inventory could be used as a basis for expanding the range of examples and resources offered within online, written and course-based guides to developing recovery stories. Narrative-based courses could ensure that their content and the way they are structured do not imply that particular elements must be present in order for the narrative to be a recovery story. Similarly, repositories collating stories as a resource may choose to use the framework as a tool to avoid potential biases. Requiring an emphasis, for example, on personal choice and responsibility within stories as criteria for inclusion may support the criticism that the use of narratives by mental health services has been co-opted in the service of neo-liberal agendas [51].

Secondly, the framework could be used to identify potential gaps in collections, highlighting areas where effort may need to be made to target specific communities to ensure their narratives are also included. This would provide a diversity metric for recovery story collections.

#### Trauma-informed practice

It is noted that the three higher-methodological quality papers which focused on narrative sequence identified a three-stage sequence only, wherein the first stage is a variation on ‘traumatic past’. This contrasts with other publications which include a sequence named ‘life before problems begin’ or similar. Considerable work is currently being undertaken on the overlap between childhood trauma and experiences of mental ill health [135, 136], and a new conceptual framework providing an alternative to diagnoses gives a central place to stories [127]. A trauma-informed approach to narrative interventions may need to take into account that there may not be a ‘before’ stage for the narrator to return to, and take care to ensure that any guidance does not presuppose the existence of such a stage.

#### Using the framework as a matching tool

Finally, the current synthesis may form the basis for a matching tool within narrative interventions. This may contribute towards increasing the confidence of practitioners in connecting individuals with the kinds of recovery narratives likely to be most beneficial to them within a particular context or time. Mental health practitioners, and recovery narratives themselves, can play a crucial role as “holders of hope” at times when an individual may be unable to construct, hold onto or believe in their own narratives of recovery [137]. However, practitioners may be limited if their conceptualisation of recovery narratives is narrow, and the impact of narratives may be limited if narrators feel no connection with the narratives they access. The multidimensional understanding of the characteristics of recovery narratives provided by this review may serve to increase the range of ways recovery can be imagined, accessed, and thus realised.

## Conclusion

This review extends the literature on mental health recovery narratives, by synthesising the various ways in which they have been characterized to produce a conceptual framework. Recovery narratives have played a vital role in establishing the recovery paradigm for survivor movements and individuals, and within mental health services and policy. Care is needed to ensure that recovery narrative interventions are used to expand the available choices within the narrating of recovery instead of curtailing them. Practitioners using narrative interventions will need to consider possible limitations of the form, structure and content of any tools offered. Mental health narrative researchers should aim to increase the diversity of populations invited to tell their recovery story.

## Supporting information

S1 File PROSPERO protocol. Review protocol registered with PROSPERO (International prospective register of systematic reviews).
(PDF)

S1 Table Data abstraction table. Showing characteristics and findings of the 45 included papers.
(XLSX)

S2 Table Summary of quantitative findings. Showing a summary of quantitative findings from two papers.
(XLSX)

S3 Table PRISMA checklist. Showing the page numbers on which Preferred Reporting Items for Systematic Reviews and Meta-Analyses (PRISMA) are reported.
(DOC)

## References

[1] Department of Health. Closing the gap: Priorities for essential change in mental health. London: Department of Health, 2014 Available from: https://assets.publishing.service.gov.uk/government/uploads/system/uploads/attachment_data/file/281250/Closing_the_gap_V2_-_17_Feb_2014.pdf

[2] Saxena S, Funk M, Chisholm D. Comprehensive mental health action plan 2013–2020. 2015. Report No.: 1020–3397 Contract No.: 7.

[3] Anthony WA. Recovery from mental illness: the guiding vision of the mental health service system in the 1990s. Psychosocial Rehabilitation Journal. 1993;16(4):11.

[4] Deegan PE. Recovery: The lived experience of rehabilitation. Psychosocial Rehabilitation Journal. 1988;11(4):11.

[5] Morrison LJ. Talking back to psychiatry: The psychiatric consumer/survivor/ex-patient movement. London: Routledge; 2013.

[6] Kirkpatrick H, Byrne C. A narrative inquiry: Moving on from homelessness for individuals with a major mental illness. Journal of Psychiatric and Mental Health Nursing. 2009;16(1):68–75. 10.1111/j.1365-2850.2008.01331.x 19192088

[7] Rhodes P, De Jager A. Narrative studies of recovery: A critical resource for clinicians. Clinical Psychologist. 2014;18(3):99–107.

[8] Davidson L, Rakfeldt J, Strauss J. The roots of the recovery movement in psychiatry: Lessons learned. New Jersey: John Wiley & Sons; 2011.

[9] LeFrançois BA, Menzies R, Reaume G. Mad matters: A critical reader in Canadian mad studies. Ontario: Canadian Scholars’ Press; 2013.

[10] Spector-Mersel G, Knaifel E. Narrative research on mental health recovery: two sister paradigms. Journal of Mental Health. 2018;27(4):298–306. 10.1080/09638237.2017.1340607 28648112

[11] Deegan P. Recovery as a journey of the heart. Psychiatric Rehabilitation Journal. 1996;19(3):91.

[12] Moran GS, Russinova Z, Gidugu V, Yim JY, Sprague C. Benefits and mechanisms of recovery among peer providers with psychiatric illnesses. Qualitative Health Research. 2012;22(3):304–19. 10.1177/1049732311420578 21900694

[13] Cronise R, Teixeira C, Rogers ES, Harrington S. The peer support workforce: Results of a national survey. Psychiatric Rehabilitation Journal. 2016;39(3):211 10.1037/prj0000222 27618458

[14] Nurser KP, Rushworth I, Shakespeare T, Williams D. Personal storytelling in mental health recovery. Mental Health Review Journal. 2018;23(1):25–36.

[15] Onken SJ, Craig CM, Ridgway P, Ralph RO, Cook JA. An analysis of the definitions and elements of recovery: A review of the literature. Psychiatric Rehabilitation Journal. 2007;31(1):9 17694711 10.2975/31.1.2007.9.22

[16] Rennick-Egglestone S, Morgan K, Llewellyn-Beardsley J, Ramsay A, McGranahan R, Gillard S, et al Mental health recovery narratives and their impact on recipients: systematic review and narrative synthesis. Canadian Journal of Psychiatry. 2019; Forthcoming. 10.1177/0706743719846108 PMC6783672 31046432

[17] Smith-Merry J, Freeman R, Sturdy S. Implementing recovery: an analysis of the key technologies in Scotland. International Journal of Mental Health Systems. 2011;5(1):11 10.1186/1752-4458-5-11 21569633 PMC3121682

[18] Smith B, Sparkes AC. Narrative inquiry in sport and exercise psychology: What can it mean, and why might we do it? Psychology of Sport and Exercise. 2009;10(1):1–11.

[19] Riessman CK. Narrative methods for the human sciences. London, Sage; 2008.

[20] Polkinghorne DE. Narrative knowing and the human sciences. New York: Suny Press; 1988.

[21] Lysaker PH, Lysaker JT, Lysaker JT. Schizophrenia and the collapse of the dialogical self: Recovery, narrative and psychotherapy. Psychotherapy: Theory, Research, Practice, Training. 2001;38(3):252.

[22] Goffman E. The Presentation Of Self In Everyday Life. New York: Doubleday And Company; 1959.

[23] Krumpal I. Determinants of social desirability bias in sensitive surveys: a literature review. Quality & Quantity. 2013;47(4):2025–47.

[24] Frank AW. Letting stories breathe: A socio-narratology. Chicago: University of Chicago Press; 2010.

[25] Frank AW. The wounded storyteller: Body, illness, and ethics. Chicago: University of Chicago Press; 2013.

[26] Ezzy D. Illness narratives: time, hope and HIV. Social Science & Medicine. 2000;50(5):605–17. 10658842 10.1016/s0277-9536(99)00306-8

[27] Swift TL, Dieppe PA. Using expert patients’ narratives as an educational resource. Patient Education and Counseling. 2005;57(1):115–21. 10.1016/j.pec.2004.05.004 15797160

[28] Kumagai AK, Murphy EA, Ross PT. Diabetes stories: use of patient narratives of diabetes to teach patient-centered care. Advances in Health Sciences Education. 2009;14(3):315 10.1007/s10459-008-9123-5 18516695

[29] McKeown J, Clarke A, Repper J. Life story work in health and social care: systematic literature review. Journal of Advanced Nursing. 2006;55(2):237–47. 10.1111/j.1365-2648.2006.03897.x 16866815

[30] Riessman CK. Ruptures and sutures: time, audience and identity in an illness narrative. Sociology of Health & Illness. 2015;37(7):1055–71. 25923981 10.1111/1467-9566.12281

[31] Woods A. The limits of narrative: provocations for the medical humanities. Medical Humanities. 2011; 37(2):73–8. 10.1136/medhum-2011-010045 22038696 PMC4281385

[32] Crossley M. Introducing narrative psychology. London: McGraw-Hill Education; 2000.

[33] Stone B. Towards a writing without power: Notes on the narration of madness. Auto/Biography. 2004;12(1):16.

[34] Saavedra J, Cubero M, Crawford P. Incomprehensibility in the narratives of individuals with a diagnosis of schizophrenia. Qualitative Health Research. 2009;19(11):1548–58. 10.1177/1049732309351110 19843964

[35] McLeod J. Narrative and psychotherapy: Sage; 1997.

[36] Gergen KJ. Therapeutic professions and the diffusion of deficit. The Journal of Mind and Behavior. 1990:353–67.

[37] McNamee S, Gergen KJ. Therapy as social construction. London: Sage; 1992.

[38] McAdams DP. The stories we live by: Personal myths and the making of the self. New York: Guilford Press; 1993.

[39] Crawford R, Brown B, Crawford P. Storytelling in therapy. Cheltenham: Nelson Thornes; 2004.

[40] White M, White MK, Wijaya M, Epston D. Narrative means to therapeutic ends. New York: WW Norton & Company; 1990.

[41] Zussman R. Autobiographical occasions: Introduction to the special issue. Qualitative Sociology. 2000 3 1;23(1):5–8.

[42] Plummer K. Telling sexual stories: Power, change and social worlds. New York: Routledge; 2002.

[43] Garrett CJ. Recovery from anorexia nervosa: A Durkheimian interpretation. Social Science & Medicine. 1996;43(10):1489–506. 8923621 10.1016/0277-9536(96)00088-3

[44] Jacobson N. Experiencing recovery: A dimensional analysis of recovery narratives. Psychiatric Rehabilitation Journal. 2001;24(3):248 11315211 10.1037/h0095087

[45] Brown W. Narratives of mental health recovery. Social Alternatives. 2008;27(4):42–8.

[46] Hall JM. Narrative methods in a study of trauma recovery. Qualitative Health Research. 2011;21(1):3–13. 10.1177/1049732310377181 20663939

[47] Ridgway P. Restorying psychiatric disability: learning from first person recovery narratives. Psychiatric Rehabilitation Journal. 2001;24(4):335 11406984 10.1037/h0095071

[48] Thornhill H, Clare L, May R. Escape, enlightenment and endurance: Narratives of recovery from psychosis. Anthropology & Medicine. 2004;11(2):181–99. 26868201 10.1080/13648470410001678677

[49] Thomas SP, Hall JM. Life trajectories of female child abuse survivors thriving in adulthood. Qualitative Health Research. 2008;18(2):149–66. 10.1177/1049732307312201 18216336

[50] Adame AL, Knudson RM. Beyond the counter-narrative: Exploring alternative narratives of recovery from the psychiatric survivor movement. Narrative Inquiry. 2007;17(2):157–78.

[51] Fisher P, Lees J. Narrative approaches in mental health: preserving the emancipatory tradition. Health. 2016;20(6):599–615. 10.1177/1363459315600774 26304707

[52] Adichie CN (2009, 7). The danger of a single story [Video file]. Available from: https://www.ted.com/talks/chimamanda_adichie_the_danger_of_a_single_story

[53] Taylor S, Leigh-Phippard H, Grant A. Writing for recovery: a practice development project for mental health service users, carers and survivors. International Practice Development Journal. 2014;4(1):1–13.

[54] SAMHSA. Share Your Story: A How-To Guide for Digital Storytelling 2018 Available from: https://www.samhsa.gov/sites/default/files/programs_campaigns/brss_tacs/samhsa-storytelling-guide.pdf.

[55] Carless D. Narrative, identity, and recovery from serious mental illness: A life history of a runner. Qualitative Research in Psychology. 2008;5(4):233–48.

[56] Grant A, Leigh‐Phippard H, Short N. Re‐storying narrative identity: a dialogical study of mental health recovery and survival. Journal of Psychiatric and Mental Health Nursing. 2015;22(4):278–86. 10.1111/jpm.12188 25655508

[57] Moher D, Liberati A, Tetzlaff J, Altman DG. Preferred reporting items for systematic reviews and meta-analyses: the PRISMA statement. Annals of Internal Medicine. 2009;151(4):264–9. 19622511 10.7326/0003-4819-151-4-200908180-00135

[58] Popay J, Roberts H, Sowden A, Petticrew M, Arai L, Rodgers M, et al Guidance on the conduct of narrative synthesis in systematic reviews. A product from the ESRC methods programme Version. 2006;1:b92.

[59] Butler A, Hall H, Copnell B. A guide to writing a qualitative systematic review protocol to enhance evidence‐based practice in nursing and health care. Worldviews on Evidence‐Based Nursing. 2016;13(3):241–9. 10.1111/wvn.12134 26790142

[60] Petticrew M, Rehfuess E, Noyes J, Higgins JP, Mayhew A, Pantoja T, et al Synthesizing evidence on complex interventions: how meta-analytical, qualitative, and mixed-method approaches can contribute. Journal of Clinical Epidemiology. 2013;66(11):1230–43. 10.1016/j.jclinepi.2013.06.005 23953082

[61] Pluye P, Robert E, Cargo M, Bartlett G, O’cathain A, Griffiths F, et al Proposal: A mixed methods appraisal tool for systematic mixed studies reviews. Montréal: McGill University 2011;2:1–8.

[62] Bal M. Narratology: Introduction to the theory of narrative. Toronto: University of Toronto Press; 2009.

[63] Garrett CJ. Recovery from anorexia nervosa: A sociological perspective. International Journal of Eating Disorders. 1997;21(3):261–72. 9097199 10.1002/(sici)1098-108x(199704)21:3<261::aid-eat6>3.0.co;2-i

[64] Anderson BA. Telling stories: personal narrative as a construction of recovery processes following psychosis: & clinical research portfolio. Doctoral dissertation, University of Glasgow. 2010.

[65] Bluffield S. 'Hopeful but fragile futures': recovery narratives in early psychosis. Doctoral dissertation, University of East Anglia. 2006.

[66] de Jager A, Rhodes P, Beavan V, Holmes D, McCabe K, Thomas N, et al Investigating the lived experience of recovery in people who hear voices. Qualitative Health Research. 2016;26(10):1409–23. 10.1177/1049732315581602 25896792

[67] Manley DS. What helps and what hinders recovery: narratives of service users and practitioners about dual diagnosis (co-existing mental health and substance misuse problems). Doctoral dissertation, University of Nottingham. 2015.

[68] McCarthy C. Social Anxiety: Personal Narratives on Journeys to Recovery. Doctoral dissertation, University of East London. 2014.

[69] Phare J. Narratives of people's everyday occupational lives following long term psychiatric hospitalisation: Auckland University of Technology; 2003.

[70] Moulding NT. Gendered intersubjectivities in narratives of recovery from an eating disorder. Affilia. 2016;31(1):70–83.

[71] O'Brien K. Art-making as a resource for the emergence of alternative personal and recovery narratives for people with an experience of psychosis. Doctoral dissertation, Canterbury Christ Church University. 2014.

[72] Sullivan PW, Starnino VR, Raster CG. In the Eye of the Beholder: Recovery and Personal Narrative. Journal of Psychosocial Rehabilitation and Mental Health. 2017;4(2):221–9.

[73] Adame AL. Recovered voices, recovered lives: A narrative analysis of psychiatric survivors’ experiences of recovery. Doctoral dissertation, Miami University. 2006.

[74] Adame AL, Knudson RM. Recovery and the good life: How psychiatric survivors are revisioning the healing process. Journal of Humanistic Psychology. 2008;48(2):142–64.

[75] Adame AL, Hornstein GA. Representing madness: How are subjective experiences of emotional distress presented in first-person accounts? The Humanistic Psychologist. 2006;34(2):135–58.

[76] Brawn P, Combes H, Ellis N. Football narratives: recovery and mental health. The Journal of New Writing in Health and Social Care. 2015;2(1):30–46.

[77] Buhagiar JPM. Psychiatric survivors and narratives of activism. Doctoral dissertation, University of East London. 2013.

[78] Carless D, Douglas K. Narrative, identity and mental health: How men with serious mental illness re-story their lives through sport and exercise. Psychology of Sport and Exercise. 2008;9(5):576–94.

[79] Matusek JA, Knudson RM. Rethinking recovery from eating disorders: Spiritual and political dimensions. Qualitative Health Research. 2009;19(5):697–707. 10.1177/1049732309334077 19380505

[80] Grinter DJ. Non-engagement in psychosis: a narrative analysis of service-users’ experiences of relationships with mental health services. Doctoral dissertation, University of Glasgow. 2012.

[81] Barnett H, Lapsley H. Journeys of despair, journeys of hope. Wellington, New Zealand: Mental Health Commission; 2006.

[82] Beiza G, Capella C, Dussert D, Rodríguez L, Águila D, Gutiérrez C, et al Institutionalized adolescents in therapy: Narratives of psychotherapy and healing from sexual abuse. Research in Psychotherapy: Psychopathology, Process and Outcome. 2015;18(2).

[83] Cohen BM. Mental health user narratives: New perspectives on illness and recovery. New York: Springer; 2008.

[84] Eli K. ‘The body remembers’: narrating embodied reconciliations of eating disorder and recovery. Anthropology & Medicine. 2016;23(1):71–85. 26982433 10.1080/13648470.2015.1135786

[85] LaMarre A. Storying “Recovery”: Exploring the Narratives of Young Women in Eating Disorder Recovery. Doctoral dissertation, University of Guelph. 2014.

[86] Shohet M. Narrating Anorexia:" full" and" struggling" genres of recovery. Ethos. 2007;35(3):344–82.

[87] Shohet M. Beyond the clinic? Eluding a medical diagnosis of anorexia through narrative. Transcultural Psychiatry. 2018;55(4):495–515. 10.1177/1363461517722467 28854861

[88] Anderson K, Hiersteiner C. Listening to the stories of adults in treatment who were sexually abused as children. Families in Society: The Journal of Contemporary Social Services. 2007;88(4):637–44.

[89] Brown W, Kandirikirira N. Report on narrative investigation of mental health recovery. Glasgow: Scottish Recovery Network. 2007.

[90] Howard J. Expecting and accepting: The temporal ambiguity of recovery identities. Social Psychology Quarterly. 2006;69(4):307–24.

[91] Vander Kooij C. Recovery themes in songs written by adults living with serious mental illnesses/thèmes de rétablissement dans des chansons écrites par des adultes ayant une maladie mentale sévère. Canadian Journal of Music Therapy. 2009;15(1):37–58.

[92] Banyard VL, Williams LM. Women's voices on recovery: A multi-method study of the complexity of recovery from child sexual abuse. Child Abuse & Neglect. 2007;31(3):275–90. 17395261 10.1016/j.chiabu.2006.02.016

[93] Harvey MR. In the aftermath of sexual abuse: Making and remaking meaning in narratives of trauma and recovery. Narrative Inquiry. 2000;10(2):291–311.

[94] Lapsley H, Nikora LW, Black RM. " Kia Mauri Tau!" Narratives of recovery from disabling mental health problems. Wellington, New Zealand: Mental Health Commission; 2002.

[95] Stott A, Priest H. Narratives of recovery in people with coexisting mental health and alcohol misuse difficulties. Advances in Dual Diagnosis. 2018;11(1):16–29.

[96] Elran-Barak RL Yael; Buchbinder Eli; Zubery Eynat. "The Road to Liberation: Metaphors and Narratives of Illness of Women recovered from Bulimia Nervosa" In: Stein D, Latzer Y, editors. Treatment and recovery of eating disorders: Nova Science Publishers; 2012.

[97] Georgaca E, Zissi A. Biographical trajectories of people struggling with severe distress: Mapping the social determinants of recovery in psychosis. Annual Review of Critical Psychology. 2017;13:1–20.

[98] Thompson RB. Client use of metaphor in selected recovery narratives. Doctoral dissertation, Kent State University. 2003.

[99] Bamberg M, Georgakopoulou A. Small stories as a new perspective in narrative and identity analysis. Text & Talk. 2008;28(3):377–96.

[100] Toth C. Identity, small stories and interpretative repertoires in research interviews. An account of market researchers' discursive positioning strategies. Journal of Comparative Research in Anthropology and Sociology. 2014;5(2):153.

[101] Ibarra H, Barbulescu R. Identity as narrative: Prevalence, effectiveness, and consequences of narrative identity work in macro work role transitions. Academy of Management Review. 2010;35(1):135–54.

[102] Jabareen Y. Building a conceptual framework: philosophy, definitions, and procedure. International Journal of Qualitative Methods. 2009;8(4):49–62.

[103] Williams CC, Almeida M, Knyahnytska Y. Towards a biopsychosociopolitical frame for recovery in the context of mental illness. British Journal of Social Work. 2015;45(suppl_1):i9–i26.

[104] Kleinman A. The illness narratives: suffering, healing, and the human condition. New York: Basic books; 1988. 10.1097/ACM.0000000000001864 28952997

[105] Grover A. Report of the special rapporteur on the right of everyone to the enjoyment of the highest attainable standard of physical and mental health. UN General Assembly 2010. Contract No.: A/65/255, para. 32.

[106] Forrest R. The implications of adopting a human rights approach to recovery in practice. Mental Health Practice. 2014;17(8):29–33.

[107] Thorne A, McLean KC. Telling traumatic events in adolescence: A study of master narrative positioning In: Fivush R HC, editor. Autobiographical memory and the construction of a narrative self: Developmental and cultural perspectives. London: Psychology Press; 2003 pp. 169–85.

[108] Repper J, Perkins R. Social inclusion and recovery: A model for mental health practice: London: Elsevier Health Sciences; 2003.

[109] Basset T, Repper J. Travelling hopefully. Mental Health Today. 2005;5:16–8. 16313154

[110] Slade M, Longden E. Empirical evidence about recovery and mental health. BMC Psychiatry. 2015;15(1):285. 26573691 10.1186/s12888-015-0678-4 PMC4647297

[111] O'Connor C, Kadianaki I, Maunder K, McNicholas F. How does psychiatric diagnosis affect young people's self-concept and social identity? A systematic review and synthesis of the qualitative literature. Social Science & Medicine. 2018;212:94–119. 30029092 10.1016/j.socscimed.2018.07.011

[112] Lysaker PH, Lysaker JT. Narrative structure in psychosis: Schizophrenia and disruptions in the dialogical self. Theory & Psychology. 2002;12(2):207–20.

[113] Lysaker P, Yanos PT, Roe D. The role of insight in the process of recovery from schizophrenia: a review of three views. Psychosis. 2009;1(2):113–21.

[114] Slade M, Blackie L, Longden E. Personal growth in psychosis. World Psychiatry. 2019;18(1):29 10.1002/wps.20585 30600621 PMC6313249

[115] Lysaker PH, Klion RE. Recovery, meaning-making, and severe mental illness: A comprehensive guide to metacognitive reflection and insight therapy. New York: Routledge; 2017.

[116] Baldwin C. Narrative, ethics and people with severe mental illness. Australian & New Zealand Journal of Psychiatry. 2005;39(11–12):1022–9. 16343305 10.1080/j.1440-1614.2005.01721.x

[117] Calhoun LG, Tedeschi RG. The foundations of posttraumatic growth: An expanded framework Handbook of posttraumatic growth. New York: Routledge; 2014 p. 17–37.

[118] Pals JL, McAdams DP. The transformed self: A narrative understanding of posttraumatic growth. Psychological Inquiry. 2004:65–9.

[119] Frazier P, Tennen H, Gavian M, Park C, Tomich P, Tashiro T. Does self-reported posttraumatic growth reflect genuine positive change? Psychological Science. 2009;20(7):912–9. 10.1111/j.1467-9280.2009.02381.x 19515115

[120] Helgeson VS, Reynolds KA, Tomich PL. A meta-analytic review of benefit finding and growth. Journal of Consulting and Clinical Psychology. 2006;74(5):797 10.1037/0022-006X.74.5.797 17032085

[121] Jayawickreme E, Blackie LE. Post‐traumatic growth as positive personality change: Evidence, controversies and future directions. European Journal of Personality. 2014;28(4):312–31.

[122] Slade M, Blackie LE, Longden E. Personal growth in psychosis. World Psychiatry. 2018. 10.1002/wps.20585 PMC6313249 30600621

[123] Leamy M, Bird V, Le Boutillier C, Williams J, Slade M. Conceptual framework for personal recovery in mental health: systematic review and narrative synthesis. The British Journal of Psychiatry. 2011;199(6):445–52. 10.1192/bjp.bp.110.083733 22130746

[124] DiClemente CC, Prochaska JO. Toward a comprehensive, transtheoretical model of change: Stages of change and addictive behaviors In: Miller WRH N., editor. Applied clinical psychology Treating addictive behaviors. New York: Plenum Press; 1998.

[125] McAdams DP, Bowman PJ. Narrating life’s turning points: Redemption and contamination In: McAdams RJ D. P., & Lieblich A. editor. Turns in the road: Narrative studies of lives in transition Washington, DC.: American Psychological Association; 2001.

[126] Russo J, Sweeney A. Searching for a rose garden: Challenging psychiatry, fostering Mad Studies. Monmouth: PCCS Books; 2016.

[127] Johnstone L, Boyle M, Cromby J, Dillon J, Harper D, Kinderman P, et al The power threat meaning framework: towards the identification of patterns in emotional distress, unusual experiences and troubled or troubling behaviour, as an alternative to functional psychiatric diagnosis. Leicester: British Psychological Society 2018.

[128] Sutton-Brown CA. Photovoice: A methodological guide. Photography and Culture. 2014;7(2):169–85.

[129] Ross LE, Gibson MF, Daley A, Steele LS, Williams CC. In spite of the system: A qualitatively-driven mixed methods analysis of the mental health services experiences of LGBTQ people living in poverty in Ontario, Canada. PloS One. 2018;13(8):e0201437 10.1371/journal.pone.0201437 30110350 PMC6093609

[130] Slade M, Amering M, Farkas M, Hamilton B, O'Hagan M, Panther G, et al Uses and abuses of recovery: implementing recovery‐oriented practices in mental health systems. World Psychiatry. 2014;13(1):12–20. 10.1002/wps.20084 24497237 PMC3918008

[131] Haroz E, Ritchey M, Bass J, Kohrt B, Augustinavicius J, Michalopoulos L, et al How is depression experienced around the world? A systematic review of qualitative literature. Social Science & Medicine. 2017;183:151–62. 28069271 10.1016/j.socscimed.2016.12.030 PMC5488686

[132] Jennings H, Slade M, Bates P, Munday E, Toney R. Best practice framework for Patient and Public Involvement (PPI) in collaborative data analysis of qualitative mental health research: methodology development and refinement. BMC Psychiatry. 2018;18(1):213 10.1186/s12888-018-1794-8 29954373 PMC6022311

[133] McEvoy PM, Nathan P, Norton PJ. Efficacy of transdiagnostic treatments: A review of published outcome studies and future research directions. Journal of Cognitive Psychotherapy. 2009;23(1):20–33.

[134] Galderisi S, Heinz A, Kastrup M, Beezhold J, Sartorius N. Toward a new definition of mental health. World Psychiatry. 2015;14(2):231–3. 10.1002/wps.20231 26043341 PMC4471980

[135] Cooke A, Basset T, Bentall R, Boyle M, Cupitt C, Dillon J. Understanding psychosis and schizophrenia. London: British Psychological Society 2014.

[136] Longden E, Sampson M, Read J. Childhood adversity and psychosis: generalised or specific effects? Epidemiology and Psychiatric Sciences. 2016;25(4):349–59. 10.1017/S204579601500044X 26156083 PMC7137611

[137] Schrank B, Bird V, Rudnick A, Slade M. Determinants, self-management strategies and interventions for hope in people with mental disorders: systematic search and narrative review. Social Science & Medicine. 2012;74(4):554–64. 22240450 10.1016/j.socscimed.2011.11.008

