# Supplementary material for: Characteristics of mental health recovery narratives: Systematic review and narrative synthesis
Source: PLoS One. 2019 Mar 28;14(3):e0214678. doi: 10.1371/journal.pone.0214678 (PMC6438542; doi:10.1371/journal.pone.0214678)
Supplement: S1 File — Review protocol registered with PROSPERO (International prospective register of systematic reviews). (PDF) [file pone.0214678.s001.pdf]

Characteristics of mental health recovery narratives as interventions to improve health and wellbeing: a systematic review and narrative synthesis

Joy Beardsley, Stefan Rennick-Egglestone, Kate Morgan, Amy Ramsay, Felicity Callard, Marianne Farkas, Rose McGranahan, Kristian Pollock, David Manley, Mike Slade

Citation

Joy Beardsley, Stefan Rennick-Egglestone, Kate Morgan, Amy Ramsay, Felicity Callard, Marianne Farkas, Rose McGranahan, Kristian Pollock, David Manley, Mike Slade. Characteristics of mental health recovery narratives as interventions to improve health and wellbeing: a systematic review and narrative synthesis . PROSPERO 2018 CRD42018090188 Available from:
http://www.crd.york.ac.uk/PROSPERO/display_record.php?ID=CRD42018090188

Review question

Aims:
To review published documents presenting original frameworks for typologies or themes of mental health recovery narratives. The ultimate aim is to increase the wellbeing and quality of life of people experiencing mental health problems, using recovery narratives as interventions.
Research question:
How have mental health recovery narratives been characterised in the literature?
Definitions:
For the purposes of this review the following definitions will apply:
Mental health problems: emotional or psychological difficulties which impact on a person or those around them. These may be described by a diagnosis made by a professional against a set of diagnostic criteria (e.g. depression, schizophrenia), or they can be self-defined without a formal label.
Recovery: the process of living as well as possible, as defined by the individual, with or without continuing to experience mental health problems.
Recovery narrative: a first-person non-fiction account of recovery from mental health problems, including elements of both adversity/struggle and of self-defined strengths/successes/survival, and which refers to events or actions over a period of time.

Searches

The search strategy has been developed in collaboration with three information specialists with expertise in systematic review searches: Emma Young (Nottinghamshire Healthcare NHS Foundation Trust) and Jane Grogan and Mary Robinson (University of Nottingham). Six approaches will be used to identify relevant studies: electronic database searching, hand-searching, citation tracking, grey literature searching, web-based searching and consultation with experts.

1. Searches of electronic databases

The following bibliographic databases will be searched:

Allied and Complementary Medicine Database (AMED) via OVID
Applied Social Science Index and Abstracts (ASSIA)
Association for Computing Machinery (ACM) Digital Library
Cumulative Index of Nursing and Applied Health Literature (CINAHL) via EBSCO
EMBASE
JSTOR
Linguistics and Language Behavior Abstracts (LLBA)
Modern Languages Association (MLA) International Bibliography via ProQuest

MEDLINE (Ovid) 1946 – present
Published International Literature on Traumatic Stress (PILOTS) Database via Proquest
PsycINFO (Ovid)
Scopus (Elsevier)
Arts and Humanities Citation Index via Web of Science.
Social Science Citation Index via Web of Science.

All databases will be searched from inception to the end of March 2018. The searches will be re-run just before the final analysis, and any further studies retrieved for inclusion.
The following search strategy has been developed for use in MEDLINE. Equivalent search terms (identified from paper meta-data where the minimum = title and abstract) will be specialised to each subsequent database.

Search strategy for MEDLINE (OVID) 1946-present:
1. exp Mental Disorders/
2. exp Behavior/
3. exp Psychological Phenomena/
4. Mental Health/
5. (Mental* or psych* or mad or madness or trauma* or distress* or ‘lived experience’).ti,ab.
6. 1 or 2 or 3 or 4 or 5
7. Mental Health Recovery/
8. Psychiatric Rehabilitation/
9. Resilience, Psychological/
10. Hope/
11. Quality of life/
12. (Recover* or transform* or resilien* or surviv* or thriv* or endur* or rebuild* or hope* or conquer* or reclaim*).ti,ab.
13. 7 or 8 or 9 or 10 or 11 or 12
14. Personal Narratives/
15. Narration/
16. Narrative therapy/
17. (narrat* or story or stories or storytelling or telling or tale* or restory* or counter-narrative* or disnarrat* or memoir* or testimon* or biograph* or autobiograph* or auto-biograph* or autoethnograph* or auto-ethnograph* or photovoice).ti,ab.
18. 14 or 15 or 16 or 17
19. (typol* or classif* or genre* or theme* or structur* or categor* or framework* or dimension* or format*)
20. 6 and 13 and 18 and 19.

The search strategy was piloted to ensure that it maintains specificity and maximises the sensitivity of the search.

2. Hand-searching
The tables of contents of the following journals will be searched from inception to the end of March 2018:

• BMJ Medical Humanities
• International Journal of Narrative Therapy and Community Work
• Journal of Medical Humanities
• Narrative Inquiry
• Narrative Inquiry in Bioethics

Other journals publishing a proportionately high number of included studies will also be hand-searched for further relevant studies.

3. Citation tracking
Studies citing included papers will be forward tracked using Scopus. Reference lists of included papers will be hand-searched.

4 Grey literature searching
Grey literature searches will be conducted using databases including Ethos, Google Scholar, BASE and OpenGrey.

5. Web-based searching
Web-based resources will be identified by internet searches including Google Scholar, ResearchGate, Academia.edu and through searching specific recovery-oriented websites (for example, Scottish Recovery Network: www.scottishrecovery.net; Boston University Repository of Recovery Resources: cpr.bu.edu/resources/recovery-repository; Social Perspectives Network: spn.org.uk).

6. Expert consultations
We will contact organisations and individuals with expertise in working with recovery narratives to identify any further key studies or frameworks (for example, people delivering "Telling Your Story" courses in Recovery Colleges; the Scottish Recovery Network's Write To Recovery initiative).

Types of study to be included

Inclusion criteria:
Studies which present or substantially advance an original framework of typologies and/or themes of mental health recovery narratives.
Exclusion criteria:
Studies of illness or mental health narratives where it is not possible to identify from title or abstract the nature of the narratives studied.

Studies where the sources are not first-person narratives, for example, narratives of family members of people with mental health problems.

Condition or domain being studied

The recovery narratives of people with lived experience of mental health problems.

Participants/population

Children, young people or adults who have experienced mental health problems (self-reported or diagnosed) at any point in their lives.

Intervention(s), exposure(s)

The personal narratives of people with lived experience of mental health problems which contain themes or details of recovery. Recovery is defined within this review as the process of living as well as possible, as defined by the individual, with or without continuing to experience mental health problems.

Comparator(s)/control

None.

Primary outcome(s)

A framework of over-arching narrative typologies (structures) and themes (content) characterising mental health recovery narratives which can be used to inform the development of future research, policy and practice within healthcare and other settings.

Improvement in the wellbeing and quality of life of people experiencing mental health problems, using

narrative-based interventions.

Secondary outcome(s)

None.

Data extraction (selection and coding)

Identified citations will be collated and uploaded to EndNote, duplicates removed and the total found recorded. The titles of all identified citations will be screened for relevance against the inclusion criteria by the lead researcher, with a randomly-selected sample (10%) double-screened for inclusion by a second researcher to establish adequate (= >90%) concordance.

Potentially relevant abstracts will then be screened by the lead researcher, with a sample (10%) double-screened for inclusion by a second researcher.
For relevant papers, full text will be obtained, and eligibility decided by the lead researcher. Studies that do not meet the inclusion criteria at this stage will be excluded, and reasons for their exclusion will be documented in the final systematic review report.
Relevant data will be collected from included studies using standardised and piloted data abstraction tables. The data extracted will include:
• Identification of the study: author, title and year
• Characteristics of the study: design, methodologies, sample size
• Study quality
• Key narrative characteristics: proposed typologies and themes.
The results of the search will be reported in full in the final report and presented in the form of a PRISMA flow diagram.

Risk of bias (quality) assessment

All included studies will be critically appraised by two independent reviewers for methodological quality using a standardized critical appraisal instrument such as the appropriate Critical Appraisal Skills Programme (CASP) 2017 Checklist.

Each study will be scored and then categorised into having either low (0-4), medium (5 -7) or high (8-10) quality. Any disagreements that arise between the reviewers will be resolved through discussion, or with a third reviewer. The results of the critical appraisal will be tabulated and reported in narrative form in the final paper, and high-quality studies will subsequently be used for a sensitivity analysis.

Strategy for data synthesis

A three-stage narrative synthesis approach will be used, based on guidance for the conduct of narrative synthesis within systematic reviews (Popay et al 2006), and conducted iteratively with stages overlapping:

1. Development of a preliminary synthesis
An initial framework of findings from included studies (identified typologies and themes of mental health recovery narratives) will be developed by the lead researcher in order to explore commonalities and differences. This will be discussed, critically reflected upon and refined by the research team.

2. Exploration of relationships within and between studies
The lead researcher will examine relationships within and across included studies in relation to the initial framework. The preliminary synthesis will be refined through a process of continuous discussion, critical reflection and feedback within the research team. Findings will be synthesised into an over-arching framework by the lead researcher.

Due consideration while synthesising results will be given to the expected high level of heterogeneity across studies in relation to e.g. methodology, participants and/or style of intervention.

3. Assessment of robustness
The robustness of the over-arching framework will be examined through interviews with relevant experts, with the framework being refined accordingly, and through appropriate processes in line with guidance from Popay et al (2006), for example critical reflection on the synthesis process by the research team; consultation with authors of primary studies (Britten et al 2002) if feasible.

Analysis of subgroups or subsets

Subgroups of papers describing the recovery narratives of people experiencing psychosis or psychosis-like symptoms will be further analysed.

Papers appraised as being of high quality will be further analysed.

Contact details for further information

Joy Beardsley
joy.beardsley@nottingham.ac.uk

Organisational affiliation of the review

School of Health Sciences, Institute of Mental Health, University of Nottingham, UK
http://www.researchintorecovery.com/NEON

Review team members and their organisational affiliations

Ms Joy Beardsley. School of Health Sciences, Institute of Mental Health, University of Nottingham, UK
Dr Stefan Rennick-Egglestone. School of Health Sciences, Institute of Mental Health, University of Nottingham, UK
Dr Kate Morgan. School of Health Sciences, Institute of Mental Health, University of Nottingham, UK
Ms Amy Ramsay. Health Service and Population Research Department, Institute of Psychiatry, Psychology and Neuroscience, King's College London
Professor Felicity Callard. Department of Psychosocial Studies, Birkbeck University
Professor Marianne Farkas. College of Health and Rehabilitation Sciences, Boston University
Dr Rose McGranahan. Unit of Social and Community Psychiatry, Blizard Institute, Barts and the London School of Medicine and Dentistry, Queen Mary University of London
Dr Kristian Pollock. School of Health Sciences, Institute of Mental Health, University of Nottingham
Dr David Manley. Nottinghamshire Healthcare NHS Foundation Trust, Nottingham, UK
Professor Mike Slade. School of Health Sciences, Institute of Mental Health, University of Nottingham

Anticipated or actual start date

05 March 2018

Anticipated completion date

31 May 2019

Funding sources/sponsors

Funders: National Institute for Health Research (Programme Grant no. RP-PG-0615-20016)
Sponsors: Nottinghamshire Healthcare NHS Foundation Trust (Ref: Slade/130817)

Conflicts of interest

None specified.

Language

English

Country
United States of America, England

Stage of review
Review_Ongoing

Subject index terms status
Subject indexing assigned by CRD

Subject index terms
Humans; Mental Health; Mental Health Recovery; Narration; Narrative Medicine; Narrative Therapy; Personal Narratives as Topic; Treatment Outcome

Date of registration in PROSPERO
07 March 2018

Date of publication of this version
12 March 2018

Details of any existing review of the same topic by the same authors

Stage of review at time of this submission

Stage	Started	Completed
Preliminary searches	Yes	Yes
Piloting of the study selection process	No	No
Formal screening of search results against eligibility criteria	No	No
Data extraction	No	No
Risk of bias (quality) assessment	No	No
Data analysis	No	No

Versions
07 March 2018
12 March 2018

PROSPERO
This information has been provided by the named contact for this review. CRD has accepted this information in good faith and registered the review in PROSPERO. CRD bears no responsibility or liability for the content of this registration record, any associated files or external websites.
